# Hetero-polycyclic aromatic systems: A data-driven investigation of structure–property relationships

**DOI:** 10.3762/bjoc.20.160

**Published:** 2024-07-31

**Authors:** Sabyasachi Chakraborty, Eduardo Mayo Yanes, Renana Gershoni-Poranne

**Affiliations:** 1 Schulich Faculty of Chemistry and the Resnick Sustainability Center for Catalysis, Technion – Israel Institute of Technology, Haifa 32000, Israel

**Keywords:** computational chemistry, database, dataset, π-conjugated, polycyclic aromatic hydrocarbons, polycyclic aromatic systems

## Abstract

Polycyclic aromatic systems (PASs) are pervasive compounds that have a substantial impact in chemistry and materials science. Although their specific structure–property relationships hold the key to the design of new functional molecules, a detailed understanding of these relationships remains elusive. To elucidate these relationships, we performed a data-driven investigation of the newly generated COMPAS-2 dataset, which contains ~500k molecules consisting of 11 types of aromatic and antiaromatic rings and ranging in size from one to ten rings. Our analysis explores the effects of electron count, geometry, atomic composition, and heterocyclic composition on a range of electronic molecular properties of PASs.

## Introduction

Polycyclic aromatic systems (PASs) – molecules made up of fused aromatic rings – are among the most prevalent classes of molecules known to humankind; indeed, it is estimated that two-thirds of known molecules contain (or are themselves) an aromatic moiety [1]. In addition to their presence in naturally occurring molecules, such as DNA and proteins, they have also been harnessed for various uses, ranging from ligands for catalysts [2], through pharmaceuticals [3], to organic semiconductors [4–5]. Despite their fundamental and applicative importance to many fields, the vast chemical space of PASs has remained largely unexplored. As a result, the relationships between the arrangement and composition of a PAS’s rings and its various molecular properties remain elusive. Revealing these relationships can deepen our understanding of these systems, as well as pave the way toward efficient and effective design of new functional PASs.

Given its breadth and complexity, a natural approach to exploring the PAS chemical space is with data-driven methods, which have proven in the last few years to be extremely successful at uncovering underlying structure–property relationships. To enable such exploration, we initiated the COMPAS Project (COMputational database of PASs), the first database dedicated to PASs and their molecular properties. The first installment, COMPAS-1 [6], contains ~35k *cata*-condensed polybenzenoid hydrocarbons (cc-PBHs) and has already enabled various directions of investigation, including by training of both interpretable machine [7] and deep learning methods [8], which led to new insights into these molecules. [Note: *cata*-condensed refers to fused PASs in which each atom participates in no more than two rings].

The second installment, COMPAS-2 [9], houses ~500k *cata*-condensed heterocyclic-PASs (cc-hPASs) comprising 11 types of aromatic and antiaromatic rings containing the heteroatoms boron, nitrogen, oxygen, and sulfur and ranging in size from four-membered to six-membered rings. Compared to the parent polycyclic aromatic hydrocarbons (PAHs), PASs containing heterocycles offer greater structural diversity as well as a much broader range of optoelectronic properties. Such molecules have been used in diverse settings, functioning as organic field effect transistors [10–12], light-emitting diodes [13–15], organic semiconductors [16–17], organic photovoltaics [1,18–22], photocatalysts [23], and biological agents for tracking or inhibition [24–25], and have also been incorporated into larger structures such as nano-hoops, in order to tune and expand their functionality [26].

Herein, we perform an in-depth analysis of the data contained within COMPAS-2, aiming to elucidate the effects of electron count, geometry, atomic composition, and aromatic nature on the molecular properties of PASs. Our goal is to delineate specific structure–property relationships that may shed light on these prevalent, yet still mysterious, compounds and serve as design principles for future PASs.

## Data

**Figure 1:**
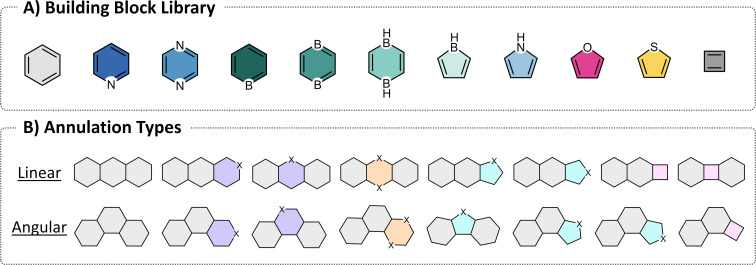
A) The building blocks used in the COMPAS-2 datasets. B) Possible annulation types formed when combining the different types of rings.

The molecules in COMPAS-2 contain 11 cyclic building blocks varying in size, composition, and aromatic character: benzene, pyridine, pyrazine, borinine, 1,4-diborinine, 1,4-dihydro-1,4-diborinine, borole, pyrrole, furan, thiophene, and cyclobutadiene (Figure 1A). These building blocks encompass 6-, 5-, and 4-membered rings with aromatic and antiaromatic character, and contain nitrogen, boron, oxygen, and sulfur atoms. Using these building blocks, we generated a chemical library of *cata*-condensed hetero-PASs (cc-hPASs) ranging in size from 3- to 10-ring systems, by combining the rings according to the annulation types shown in Figure 1B. The number, type, and position of the individual building blocks were determined randomly to avoid biasing the data and to increase the likelihood of sampling previously unstudied cc-hPAS structures. Further details on the structure enumeration and data generation are reported elsewhere [9].

It is important to note that in constructing the COMPAS-2 dataset, we opted to maintain equal percentages of the different types of heterocycles (~10% of each type). This was done to avoid biasing the construction of molecules towards specific motifs. However, because there are multiple types of B-containing and N-containing heterocycles, and because some of them contain more than one heteroatom, this resulted in an unequal distribution of the heteroatoms themselves. Thus, due to the design of the dataset construction, the relative prevalence is approximately 6:4:1:1 for B:N:O:S.

COMPAS-2 comprises two datasets – COMPAS-2x and COMPAS-2D. The former contains geometries and molecular properties obtained at the GFN1-xTB [27] level for 524,392 unique cc-hPASs. The latter is a representative subset of the former, containing 52,000 cc-hPASs with geometries optimized and properties obtained at the CAM-B3LYP/def2-SVP [28–33] level, including the D3 dispersion correction [29] by Grimme with Becke-Johnson damping [30]. We used the DFT-calculated dataset to generate fitting functions, such that all xTB-generated data was corrected to near DFT-level accuracy [9]. It is these corrected data that we use in this report to analyze the structure–property relationships of the cc-hPASs.

## Results and Discussion

**Figure 2:**
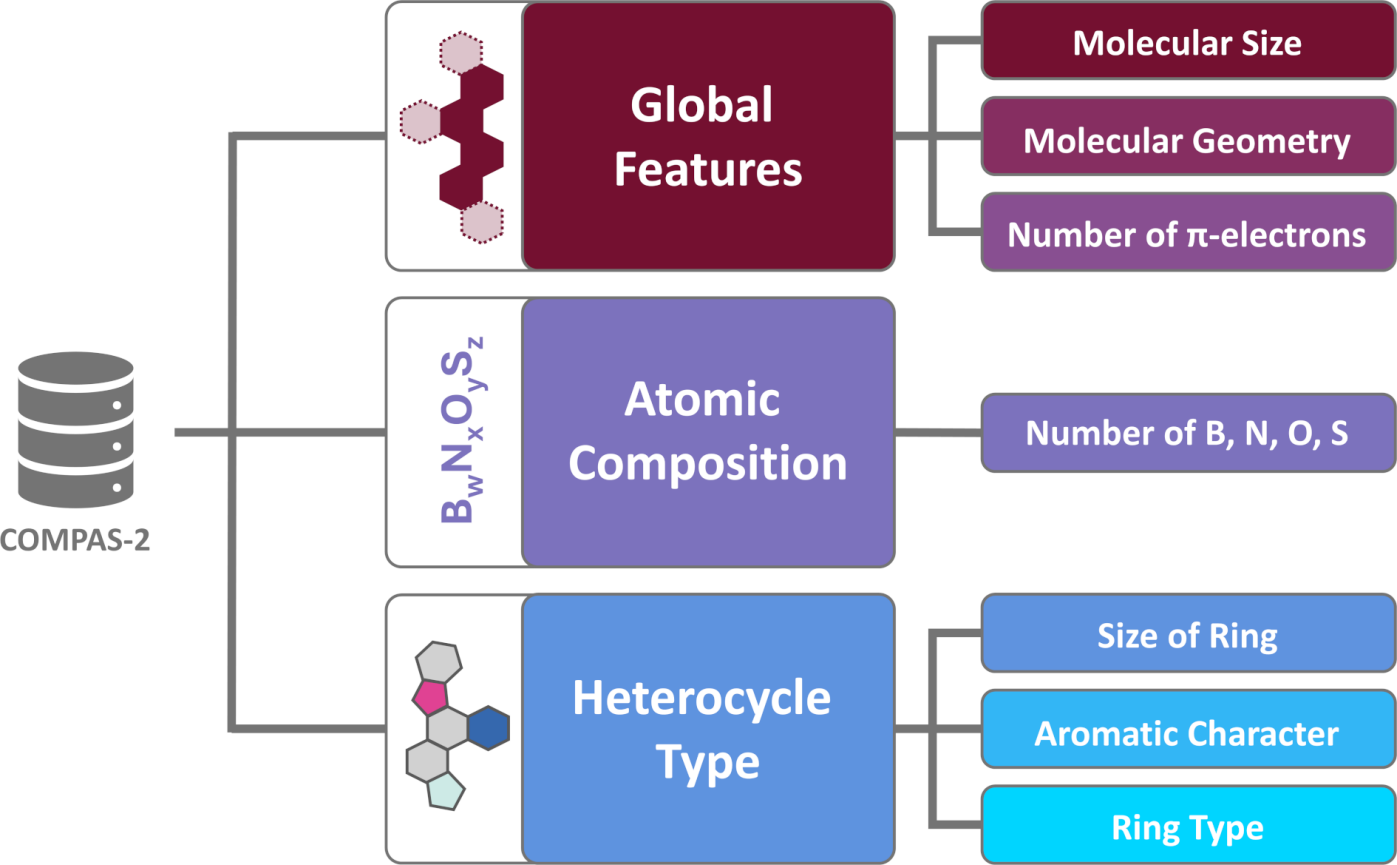
Roadmap of the work described in this article. Three main criteria are defined in increasing structural information content. For each criterion, we note the specific features that are studied in this work. For each of these, we investigate the effect of the feature on the set of molecular properties.

At first glance, the chemical space of PASs may appear to be quite homogenous. After all, the molecules share certain structural features, such as their multi-ring structures, rigidity, and π-conjugation. Nevertheless, simply by changing the combination of the individual building blocks (i.e., rings), we obtain molecules with varying sizes, geometries, atomic compositions, and aromatic character. In such multi-faceted data, it can be difficult to ascertain which structural features determine the different molecular properties. Therefore, we designed the current study with an aim to chart a clear path through this chemical space, and we present our findings along these same lines (as illustrated in Figure 2). In the first section, we provide context, giving a short comparison between the data contained within COMPAS-2 [9] and COMPAS-1 [6]. The second, third, and fourth sections then present analyses of the data, each focused on different structural aspects: global electronic and geometric features, atomic composition, and heterocyclic composition. A roadmap of the article structure is shown in Figure 2.

### Comparison between COMPAS-1 and COMPAS-2

To obtain a better overview of the COMPAS-2 chemical space and to study the effects of including these new components, we first compare the cc-PBHs in COMPAS-1 to the cc-hPASs in COMPAS-2, in terms of their shape diversity and molecular properties. As mentioned above, COMPAS-1 houses PBHs, molecules comprising only one type of ring – the aromatic, six-membered, carbon-based benzene.

**Figure 3:**
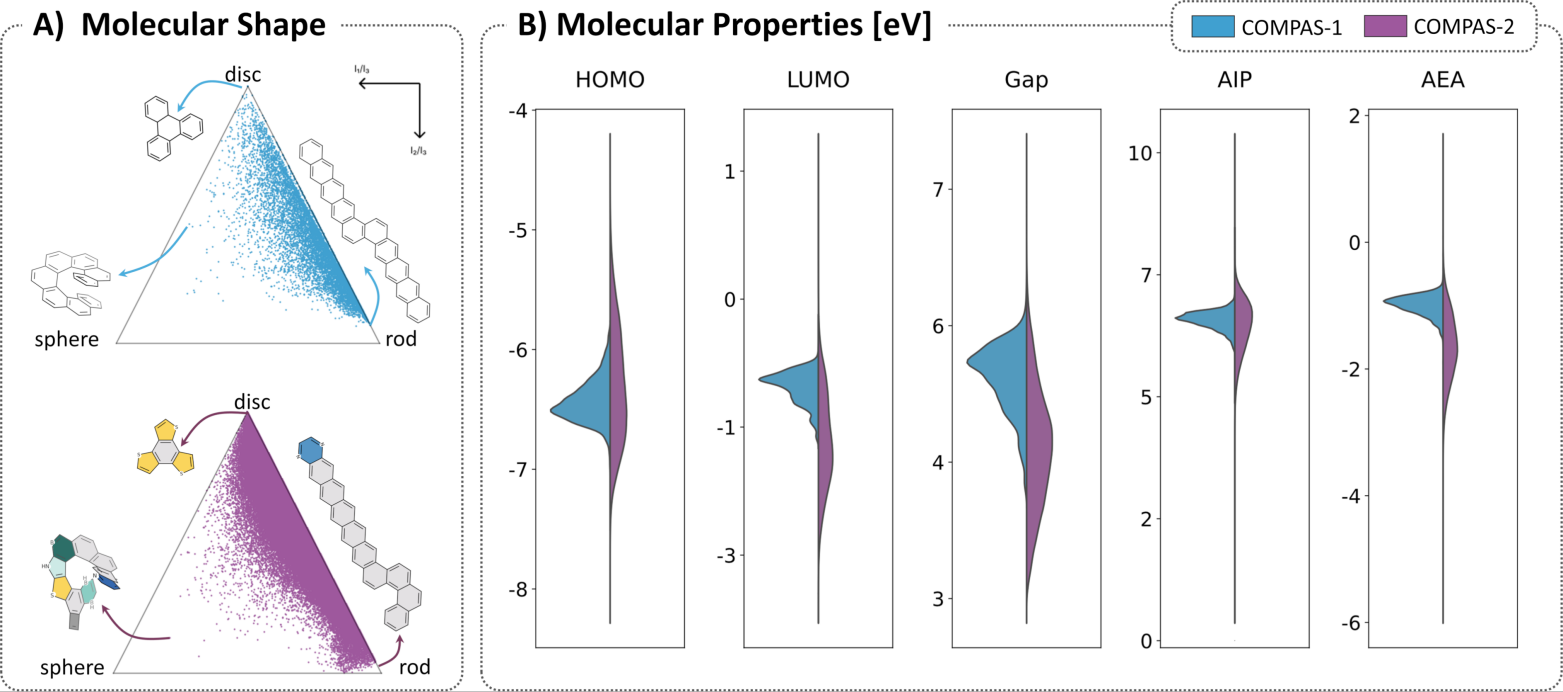
Comparison between COMPAS-1 (blue) and COMPAS-2 (purple). A) Principal Moments of Inertia shape distribution, all molecules sorted according to their normalized principal moments of inertia (I_n_, *n* = 1–3), with I_1_ < I_2_ < I_3_. B) Molecular properties (all reported in eV): HOMO, LUMO, AIP, and AEA.

The principal moments of inertia (PMI) plots in Figure 3A show that the two datasets have similar tendencies to form “rod” and “disc”-like structures (i.e., 1D or 2D, respectively). Because some of the building blocks contained in the COMPAS-2 library can only annulate linearly (specifically, cyclobutadiene, pyrazine, 1,4-diborinine, 1,4-dihydro-1,4-diborinine), this dataset shows a greater density of structures close to the “rod” vertex and along the “rod/disc” edge of the PMI plot. COMPAS-1 molecules have a higher tendency to form angular annulations and branching points and therefore we observe the increased density closer to the “disc” corner. Both datasets have very few structures close to the “sphere”-like vertex, which represents 3D geometries, i.e., non-planar molecules. For PASs, it is unlikely to find actual “sphere”-like molecules, as the individual building blocks have rigid and planar geometries and fusing such components together in a *cata*-condensed fashion is unlikely to generate molecules with a spherical structure. Rather, for our dataset, the 3D-type molecules are expected to be those with helical structure. Indeed, as we highlight in Figure 3A, the polycyclic molecules that inhabit the spherical corner of the plot are those that have helical structures, and this is common to both data sets. In other words, the comparison between the two datasets demonstrates that increasing the diversity of conjugated cyclic building blocks does not have a notable impact on the relative distribution of molecular shapes.

In contrast to their relatively high geometric similarity, the molecular properties of the two datasets vary substantially. We compared the distributions of five molecular properties: highest occupied molecular orbital (HOMO) energy, lowest unoccupied molecular orbital (LUMO) energy, HOMO–LUMO gap (Gap), adiabatic ionization potential (AIP), and adiabatic electron affinity (AEA). As shown in the violin plots in Figure 3B, for all properties, the distribution of the COMPAS-1 molecules (light blue) is contained within the distribution of the COMPAS-2 molecules (purple). In other words, the expansion of the building block library widens the property distributions towards both higher and lower energies, providing access to functional molecules with different (opto)electronic properties. For example, while the HOMO energies of the COMPAS-1 PBHs range between −7 and −6 eV, the HOMO energies for the COMPAS-2 cc-hPAS cover the range from −8 to −4.5 eV, a widening of 3.5 eV. Similarly, the range of LUMO energies expands substantially, from −0.8 to −1.8 eV in the cc-PBHs to +0.2 to −3.5 eV in the cc-hPASs, with a larger tendency towards lower-lying LUMOs than in the PBHs. The remaining properties show similar expansions of property ranges.

Overall, the comparison between the two datasets demonstrates that the cc-hPASs are structurally similar to cc-PBHs, notwithstanding the higher tendency of the COMPAS-2 molecules towards linear annulations (due to the types of building blocks used). In contrast, their electronic properties cover much broader ranges, which is what makes them so promising as functional compounds. However, to what extent each type of building block affects the properties, and whether these effects are due solely to the presence of the heteroatoms or to the aromatic nature of heterocycles are among the questions we aim to answer in the subsequent sections.

### Influence of global structural features

In this section, we investigate the effect of global structural features on the set of electronic properties detailed above (HOMO, LUMO, Gap, AIP, AEA). At this lowest resolution analysis, we aim to ascertain to what extent the overall molecular size and geometry determines molecular properties.

#### Molecular size

**Figure 4:**
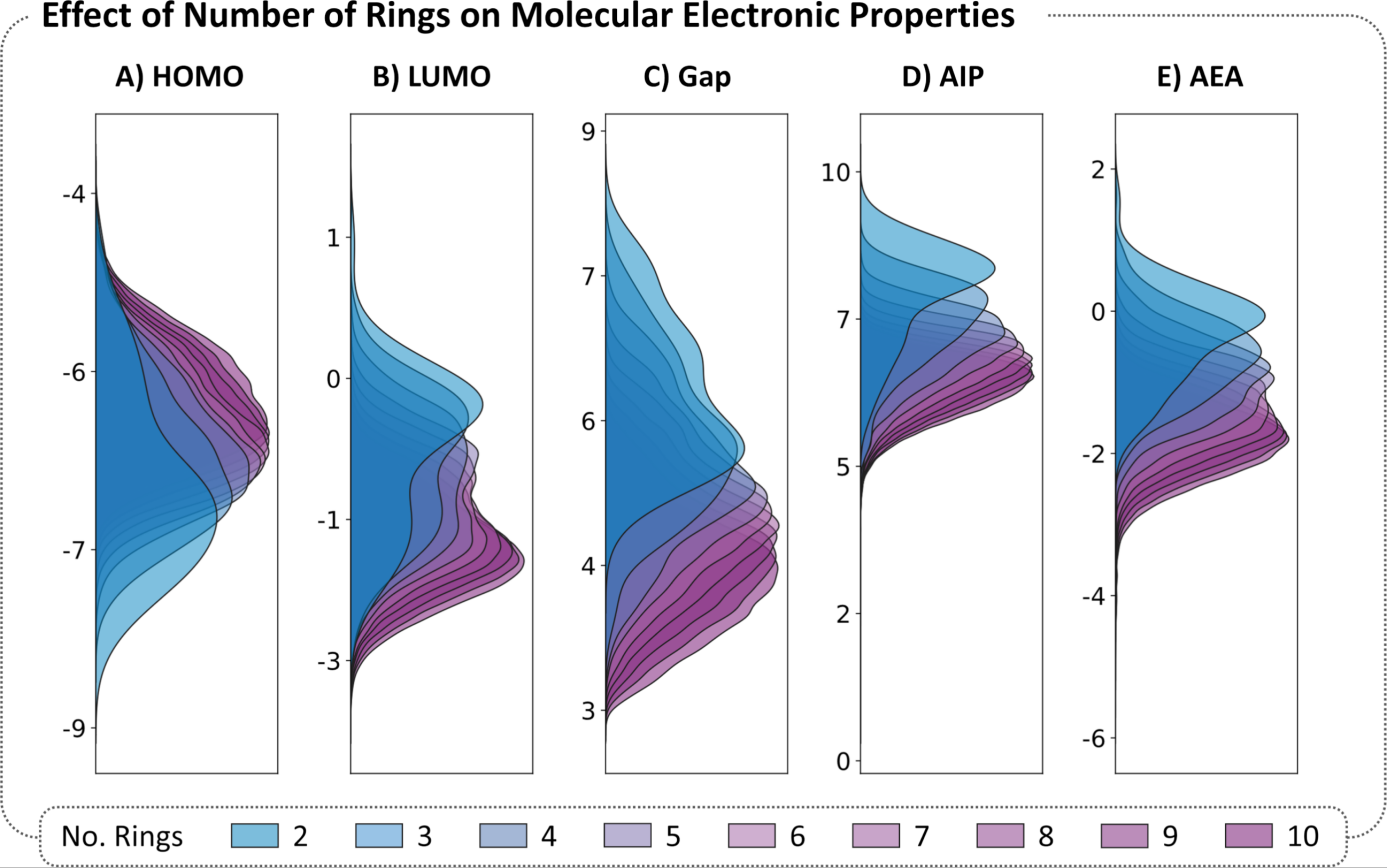
Distributions of electronic properties of the dataset. KDE plots of HOMO, LUMO, Gap, AIP, and AEA colored according to the number of rings in the molecule. The KDEs are normalized such that the area under the curve is equal to 1 for each family.

The molecular size of cc-hPASs may be evaluated in various ways – e.g., by the total number of atoms, total number of rings, total number of electrons (or specifically π-electrons). In our view, the number of rings is the simplest and most intuitive metric; it has the added benefit of revealing trends while still rendering a manageable number of groups. Therefore, we chose this descriptor and plotted the kernel-density estimates (KDEs) of the distributions of the five properties described above, colored according to the number of rings in the molecule (Figure 4). In all cases we observe “drifts”: for the HOMO, the values become less negative as the molecules increase in size; for the LUMO, Gap, AIP and AEA, the values become smaller or more negative as the molecules increase in size. These trends align with the commonly known effect in polyenes and annulenes, whereby increasing conjugation causes the HOMO to be raised, the LUMO to be lowered, and the Gap to be reduced. The differences between consecutive groups become smaller as the molecules grow in size, which is consistent with the 1/*n* relationship reported for other polycyclic systems [34].

#### Molecular geometry

In our previous work on cc-PBHs, we observed a similar correlation to size, however, our data-driven analysis revealed that the underlying source of the correlation is not just the molecular size, *per se*, but rather the formation of linear stretches (substructures that are linearly annulated) [6]. Accordingly, the longer the linear stretch, the higher the HOMO, the lower the LUMO, etc., and the apparent size dependency arises simply because larger molecules have more opportunities to create longer linear stretches.

**Figure 5:**
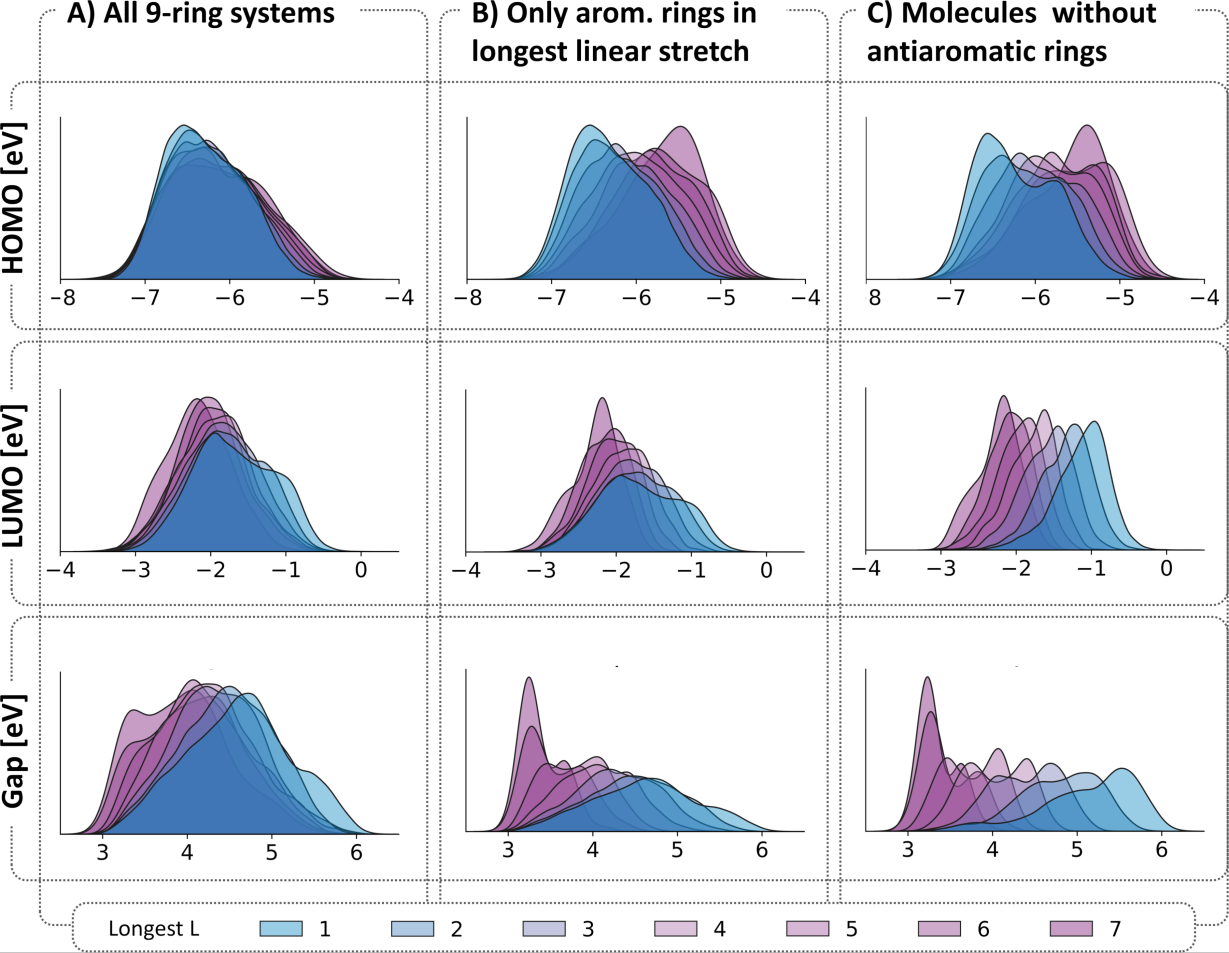
KDE distributions of the HOMO, LUMO, and Gap separated and colored by the longest L for: A) all 9-ring molecules; B) all 9-ring molecules that do not have an antiaromatic moiety in the longest linear stretch; C) all 9-ring molecules that do not contain any antiaromatic moiety. The KDEs are normalized such that the area under each curve is 1.

The scatter plot of the HOMO/LUMO space showed that a similar trend does exist for COMPAS-2, albeit weaker than the COMPAS-1 case (see section 1.1 in Supporting Information File 1 for further details). To investigate this further, and to avoid the size-dependency issue, we focused only on the 9-ring systems in the dataset. In this collection of 152,121 molecules, all molecules have the same number of rings but differ in their annulations and composition and therefore have varying numbers of atoms and π-electrons. We plotted the KDE distributions of the HOMO, LUMO, and Gap for this subset of data, colored according to the longest linear stretch in the molecules (Figure 5A). Note that, for cc-hPASs, a linear annulation is defined as three consecutive rings having an angle of 180° between the ring centroids; any angle that is not 180° is considered to be an angular annulation (see Figure 1B). Indeed, although a trend may be observed, it is not nearly as pronounced as the trend we observed for the cc-PBHs in COMPAS-1 [6]. This led us to hypothesize that the presence of antiaromatic moieties in the linear stretch (cyclobutadiene and/or 1,4-dihydro-1,4-diborinine) may be distorting the results. In other words, perhaps the effect is only relevant to linear stretches of aromatic rings. To verify this, we identified within the same molecules the longest linear stretch comprising only aromatic rings. These distributions (shown in Figure 5B) do indeed show a clearer trend, but it is still weaker than the cc-PBHs. Therefore, in the next step, we plotted the distributions of all 9-ring molecules containing only aromatic building blocks (i.e., have no cyclobutadiene, borole, or 1,4-dihydro-1,4-diborinine moieties; a collection of 127,019 molecules). In this case (Figure 5C), the stratification of the data did become more pronounced, indicating that antiaromatic rings mask the longest linear stretch effect. Overall, these results show that the longest linear stretch effect does generalize from cc-PBHs to cc-hPASs, but it is most significant for cc-hPASs that comprise only aromatic rings. This observation aligns with previous experimental work from the groups of Vollhardt. Miao, and Xia, who studied diareno-fused cyclobutadienes and found similar trends [35–38].

In addition to this structural feature, we also examined the effects of the number of branching points and deviation from planarity. Neither of these structural features showed any meaningful trend (further details are provided in sections 1.2 and 1.3 of Supporting Information File 1).

#### π-Electron count

In contrast to COMPAS-1, COMPAS-2 contains both molecules with a (4*n* + 2) π-electron count and molecules with a 4*n* π-electron count, allowing us to study the difference between formally Hückel aromatic and formally Hückel antiaromatic PASs. We note in this regard that the ‘Hückel Rule’ (a term that was actually introduced by Doering) [39] was originally developed solely for monocyclic systems, but was later extended by Vol’pin to *cata*-condensed polycyclic systems [40].

**Figure 6:**
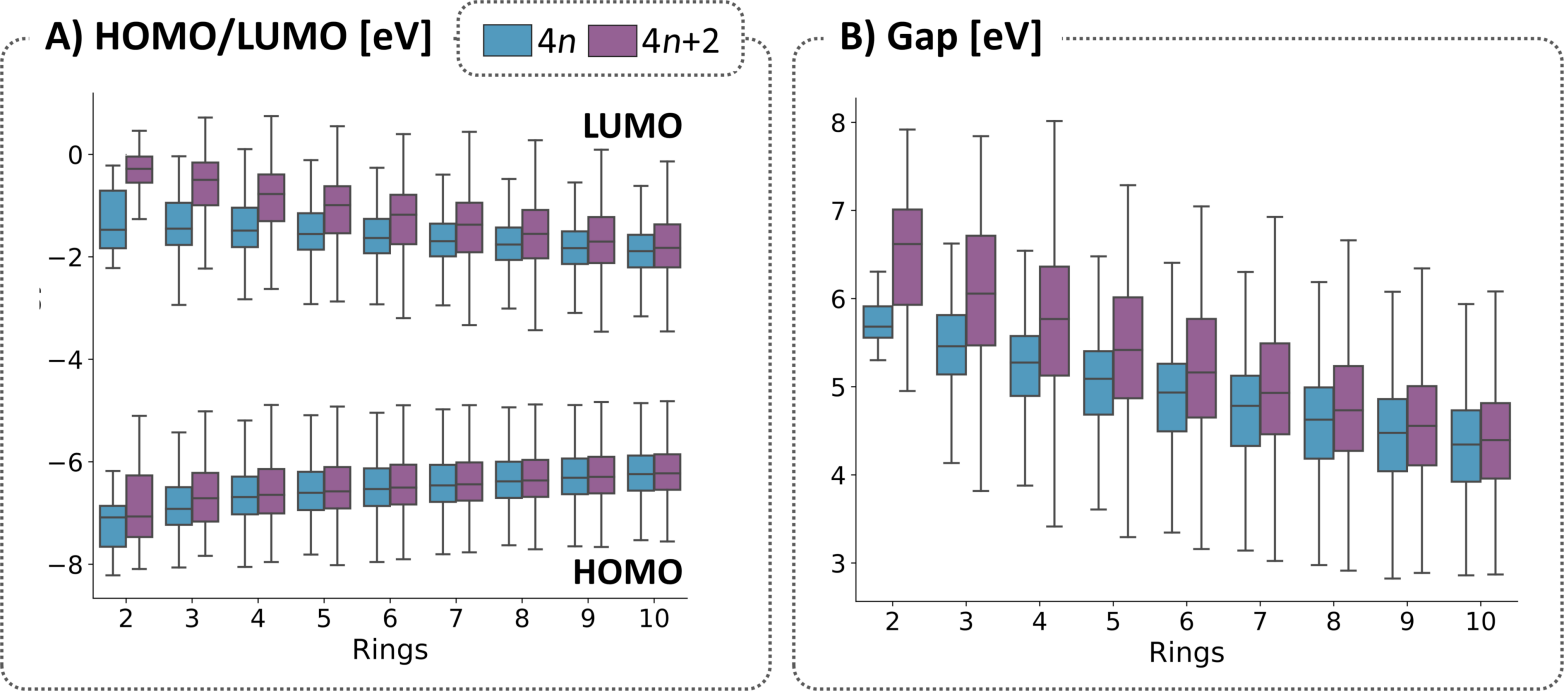
Distributions of molecular properties for 4*n* and (4*n* + 2) π-electron count systems, divided by the number of rings for: A) HOMO and LUMO energies and B) Gaps.

It is generally assumed that aromatic molecules are characterized by excess stability and a large Gap, while antiaromatic molecules are less stable and have smaller Gaps. To investigate whether this assumption holds true for cc-hPASs, we plotted the distributions of several properties for the two subsets of molecules, separated by size. As seen in Figure 6A, the distributions of the HOMO and LUMO values are both higher for the (4*n* + 2) systems than for the (4*n*). However, these differences diminish at different rates: for the HOMOs, the two distributions become essentially indistinguishable at 4-ring systems, whereas for the LUMOs it is only at 10-ring systems that the values attain parity. Unsurprisingly, the (4*n* + 2) systems show higher Gap values (Figure 6B), however, the difference consistently diminishes until it is negligible for 10-ring systems. (For further analysis based on this criterion, including molecular stability, see section 1.4 of Supporting Information File 1.)

### Influence of atomic composition

The incorporation of different atoms is a well-known strategy for modulating the frontier molecular orbitals of π-conjugated systems. For example, it has been empirically observed, and can also be rationalized with molecular orbitals-based considerations, that lone-pair bearing heteroatoms such as oxygen raise the HOMO level [41–42], while boron lowers the LUMO level [43]. COMPAS-2 provides, for the first time, the possibility to substantiate these observations in a data-driven manner and, perhaps, to extract quantitative assessments of these effects. In this section, we study the effects of the presence and number of different heteroatoms on the electronic properties of the molecules in COMPAS-2.

We first visualized the distribution of the various types of heteroatoms across the property space by generating a series of scatter plots (HOMO versus LUMO) and coloring each plot according to the number of heteroatoms of a certain type (section 2 of Supporting Information File 1). These plots (Figure S6 in Supporting Information File 1) made it readily apparent that the B atoms unsurprisingly accumulate in the regions of lower LUMO value and, to a lesser extent, higher HOMO values. The N, O, and S atoms appear to be more evenly distributed over the property space, however, certain areas can be identified with slightly higher populations of heteroatom-rich PASs.

**Figure 7:**
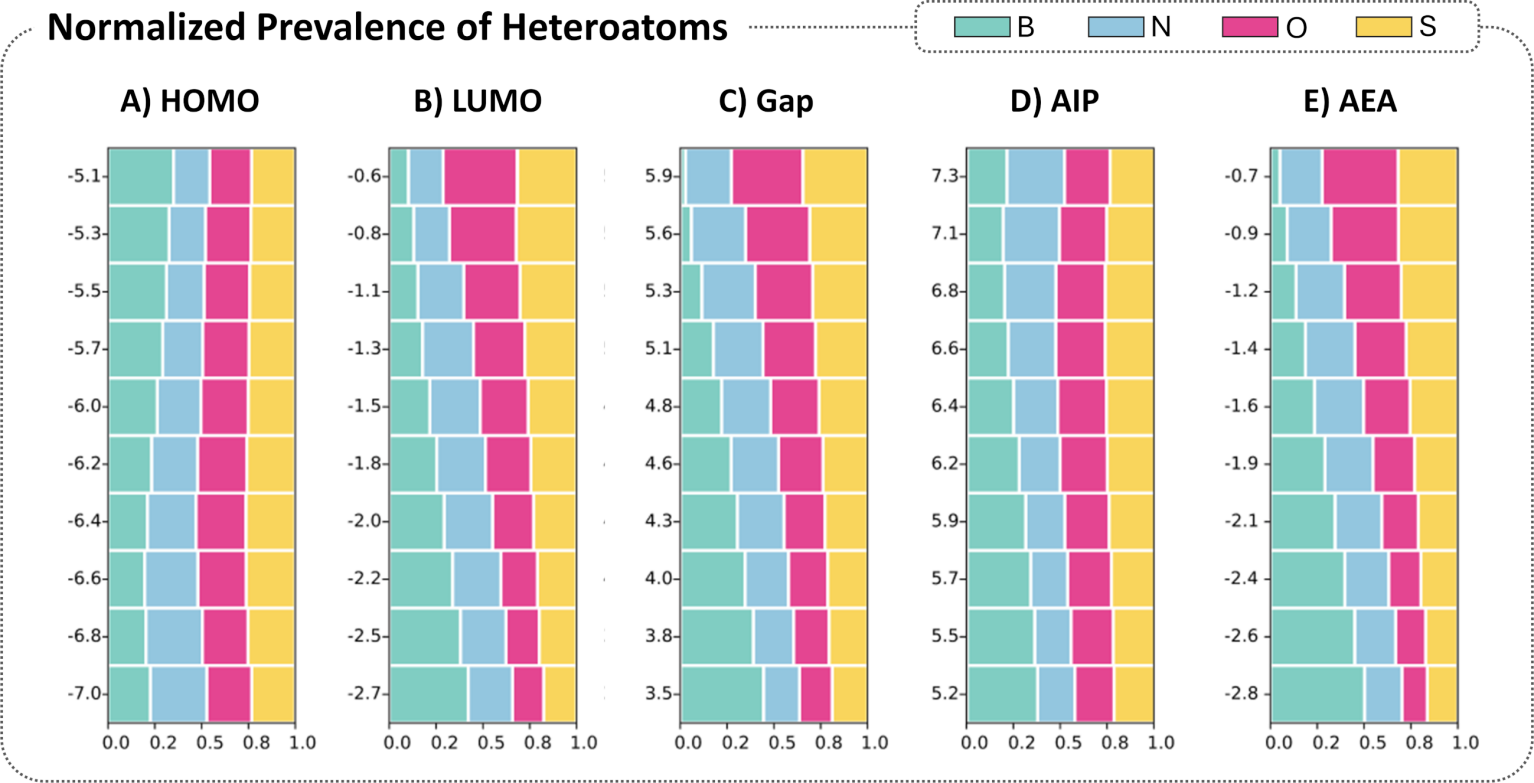
Stacked histograms showing the prevalence of the different heteroatoms across different areas of the property space, for A) HOMO, B) LUMO, C) Gap, D) AIP, and E) AEA. The colors of each block correspond to the color-coded heteroatoms shown at the top. The size of each block represents the fraction of that heteroatom out of the overall number of heteroatoms in that respective bin. The sum of all heteroatoms is 1 for each bin. Molecules below and above the 0.01 and 0.99 percentiles, respectively, were discarded as not statistically meaningful.

To explore this further, we divided each property into ten evenly spaced sections and binned all the molecules in each respective section (as before, this analysis focused on the 9-ring systems, to avoid any size-dependency artifacts). For each bin, we plotted the relative prevalence of the various heteroatoms as a stacked histogram (Figure 7), where the different colored blocks represent the different heteroatoms and the sum of all blocks in each bin is equal to 1. The size of each block represents the likelihood of a heteroatom from this bin being a certain type. As we noted in the Data section, the total numbers of heteroatoms are not equal (the ratios of B:N:O:S atoms are approximately 6:4:1:1). Therefore, each block was normalized according to the relative prevalence of the heteroatom in the dataset, which allows for a more straightforward comparison between different heteroatoms, as well as for the same heteroatom across the property range.

Figure 7 shows that the likelihood of finding O and S atoms is relatively uniform across the HOMO range, while it decreases for N and increases for B as the HOMO values rise. The trends are more pronounced for the LUMO: the B atoms are clearly most prevalent at the lower LUMO levels and steadily decrease towards the higher LUMO levels, concurrent with a steady rise in the likelihood of O and S atoms, while the N likelihood remains rather uniform. The trends become even more pronounced in the plot of the Gap, as it is a sum of the HOMO and LUMO complementary effects. For the Gap and AIP, an increase in N towards higher values can be noted. For the AEA, the B clearly dominates the lower values, while again N, S, and especially O show an increase towards the higher values. We note that the relative uniformity of the N prevalence across the various property ranges could be due to contradicting effects of the different types of N-containing rings and does not necessarily imply that N does not have a strong impact on the properties. Conversely, the prevalence of B at certain property values does not mean that all B-containing systems have similar effects; it could be that one or more B-containing systems have stronger effects that dominate. These questions are addressed in subsequent sections.

### Influence of heterocycle type

In this section, we focus on the character and type of the rings comprising the cc-hPASs, going from the broader perspective (size) to a more detailed view (aromatic/antiaromatic) and finally to the specific type of ring.

#### Size of ring

In the broadest sense, without analyzing their specific composition or character, the individual building blocks in our library may be categorized according to their sizes. To study the effect on the molecular properties, we plotted the KDE distributions of the various properties, separated by the number of 4-, 5-, and 6-membered rings, respectively. We observed that the sizes of the individual rings do not have an inherent effect on the electronic properties (see Supporting Information File 1, section 3.1 for further details).

#### Aromatic character of the rings

**Figure 8:**
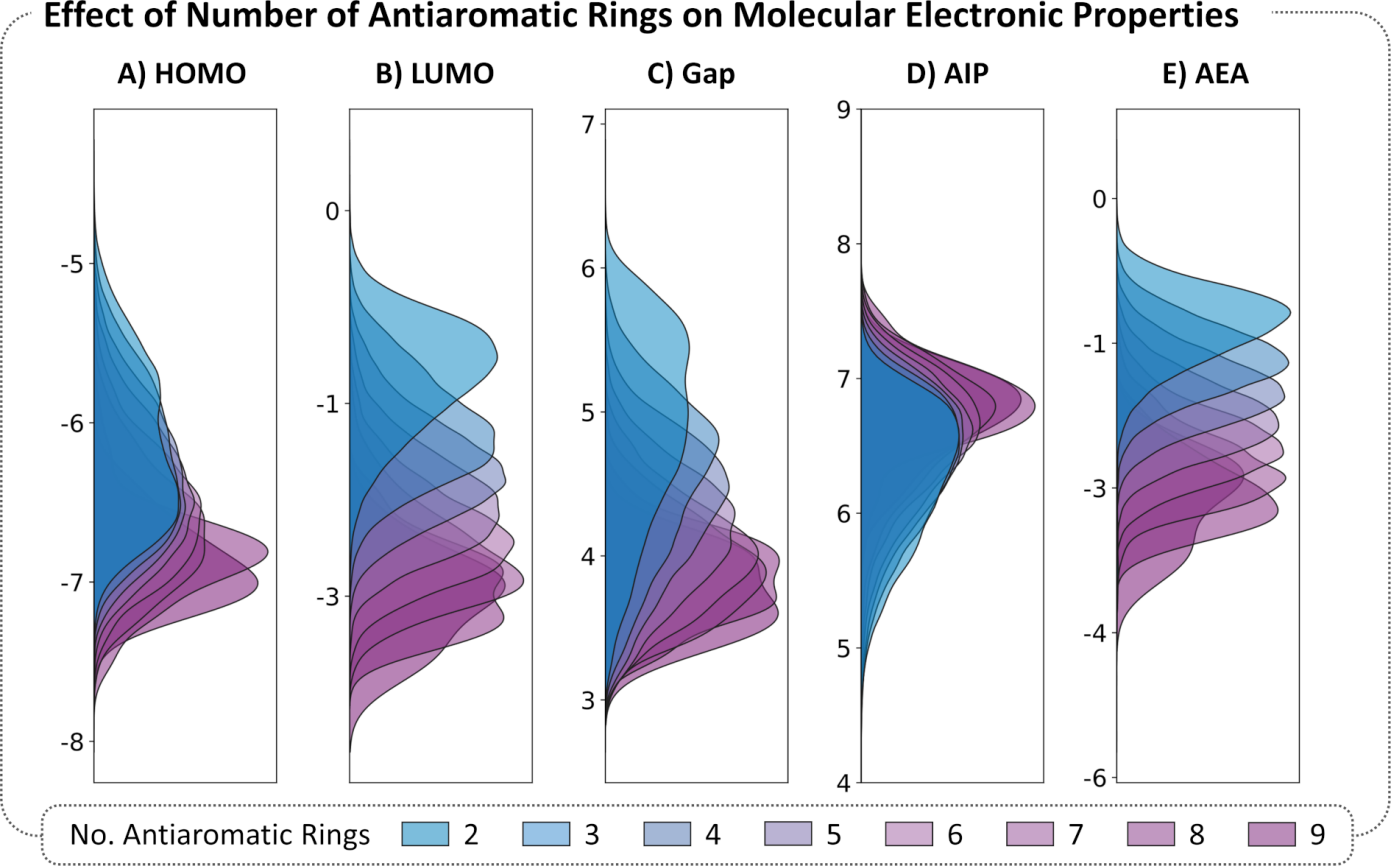
Distributions of electronic properties of the dataset. KDE plots of A) HOMO, B) LUMO, C) Gap, D) AIP, and E) AEA colored according to the number of antiaromatic rings in the molecule. The KDEs are normalized such that the area under the curve is equal to 1 for each family.

The rings can be further classified as Hückel aromatic [(4*n* + 2) π-electrons] or antiaromatic [(4*n*) π-electrons)]. To study the relationship between the number of rings of each type and the molecular properties, we plotted the distributions of the five molecular properties, separated by the number of antiaromatic rings (Figure 8). Once again, to circumvent the size-dependency issue (see section "Molecular size" above), we analyzed only the molecules containing 9 rings (a subset of 152,121 molecules). All the properties showed a definite trend, although it appears to be strongest for the LUMO and Gap and smallest for the AIP. Overall, molecules with a higher number of antiaromatic moieties show lower HOMOs, lower LUMOs, lower Gaps, and stronger electron affinity – regardless of the specific type of rings that are contained in the molecule. However, we note that two of the three antiaromatic rings in our library are B-containing heterocycles. As shown above, boron also has a strong effect on the molecular properties. Thus, it is unclear whether the apparent trends here stem from the boron atom or from the antiaromatic character of the building blocks. This will be addressed in the subsequent section. (Additional analysis based on this descriptor is provided in Supporting Information File 1, section 3.2.).

#### Specific identity of heterocycle

The previous analyses revealed two relationships: lower LUMO values were shown to correspond to both the presence of boron-containing rings and to the presence of antiaromatic rings. However, two-thirds of our antiaromatic building blocks *are* boron-containing rings (borole, 1,4-dihydro-1,4-diborinine). Thus, it is not clear whether these trends are due to the identity of the heteroatom or to the nature of the ring. To answer this question, we investigated the influence of each individual building block.

**Figure 9:**
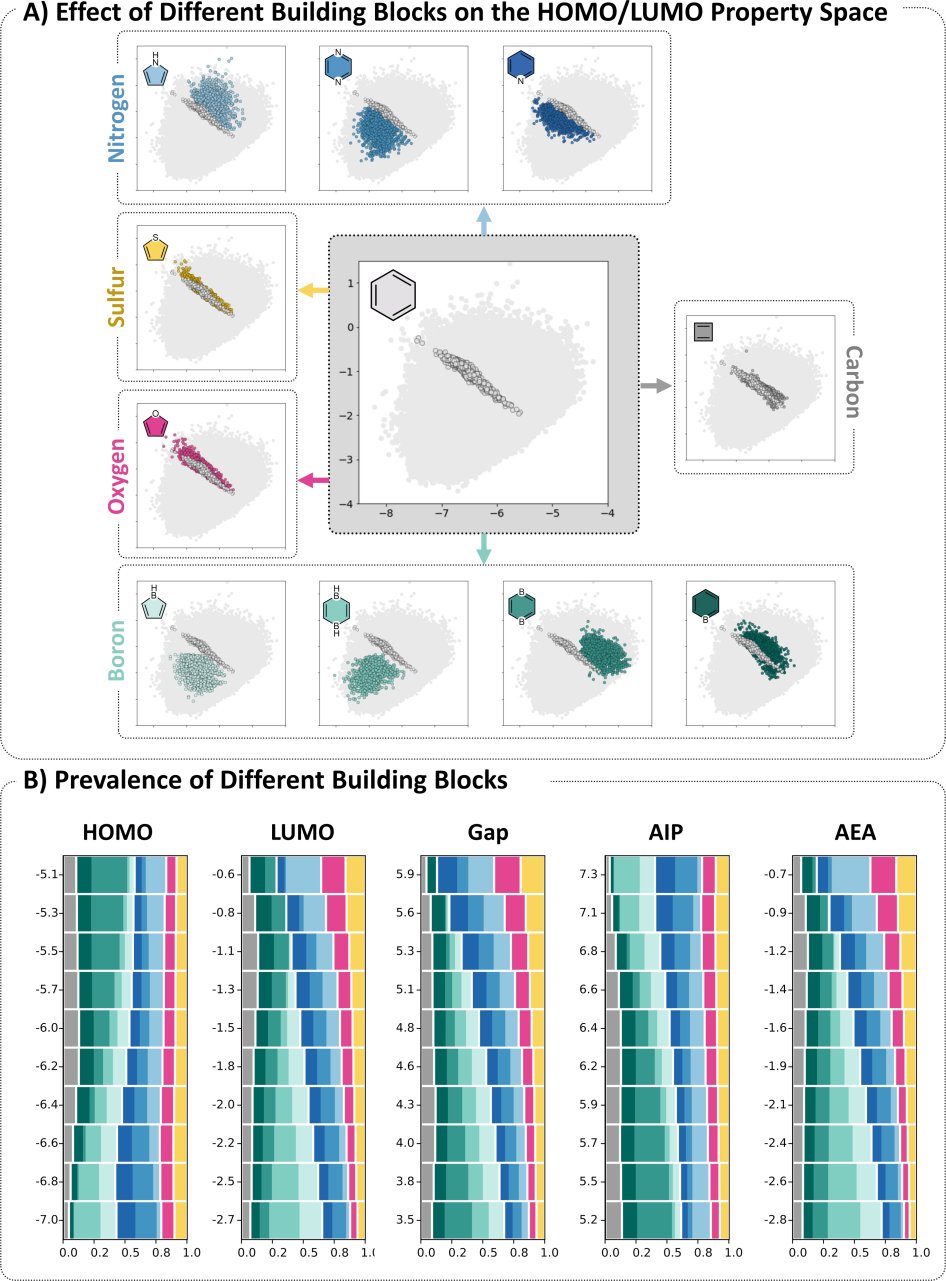
A) Scatter plot of the HOMO (*x*-axis) and LUMO (*y*-axis) values of the molecules in COMPAS-2. In each plot, the molecules containing only benzene are colored in gray and the molecules containing only benzene and a single other type of building block are colored according to the color-coded library. B) Stacked histograms showing the prevalence of the different building blocks for HOMO, LUMO, Gap, AIP, and AEA. The colors of each block correspond to the color-coded molecules. The size of each block represents the fraction of that building block out of the overall building blocks (excluding benzene). The sum of all building blocks is 1 for each bin. Molecules below and above the 0.01 and 0.99 percentiles, respectively, were discarded as not statistically meaningful. Note: for the plot of borinine, 5 outlier data points were removed following visual inspection, which determined that these molecules did not optimize correctly.

Figure 9A shows scatter plots of the HOMO versus LUMO, each colored according to the presence of a specific type of heterocycle. To avoid ambiguity, only molecules that contain benzene and the heterocycle highlighted in the respective plot are colored (i.e., molecules that contain mixtures of heterocycles are not colored). This is to ensure that our focus is on the effect of one specific heterocycle at a time.

Several observations can be made based on Figure 9, which are best demonstrated in comparison to the cc-PBH data (i.e., using this data as a “baseline”). Thus, each different types of building block are plotted together with the cc-PBHs contained in COMPAS-2 (light gray circles). First and foremost, we note that molecules containing the same heterocycle tend to cluster in the same region of the HOMO/LUMO space, rather than be dispersed over the entire space. Secondly, we note that the shape and breadth of the property space covered differs noticeably. Furan, thiophene, and cyclobutadiene all cover a similar region of the property space as the baseline PBHs, which is a relatively small swath that shows a linear relationship – meaning, molecules with higher HOMOs have lower LUMOs and vice versa. In contrast, for all the B- and N-containing heterocycles, the respective regions are quite broad and without a well-defined shape, meaning that it is possible to find molecules with different combinations of low/high/mid-range HOMO and LUMO values within the region. Overall, it is apparent that the significant increase in property space over the COMPAS-1 baseline (Figure 3) is due mostly to the B- and N-containing heterocycles, or to heterogeneous mixing of different types of heterocycles, which suggests a cumulative effect of incorporating different types of building blocks (for additional details on the coverage of property space, see section 4 of Supporting Information File 1).

Having several types of N-containing and B-containing heterocycles enables us to further explore the behavior of these systems. For the B-containing heterocycles, we observe that the two aromatic rings (borinine and 1,4-diborinine) both shift the distribution to the right of the PBH baseline, towards higher HOMO values, while remaining in a similar range of LUMO values. In contrast, the two antiaromatic rings (borole and 1,4-dihydrodibornine) both shift the distribution to the left and downwards, towards lower HOMO and lower LUMO values. This sheds new light on our previous observations and the question we posed at the beginning of this section, regarding the “boron-effect” and the effect of antiaromatic rings. Namely, these plots elucidate that the LUMO-lowering effect of the boron atoms is not a general rule for boron, nor is it a general rule for antiaromatic components (cyclobutadiene, another antiaromatic building block, does not exhibit the same effect). Rather, it stems from the presence of boron atoms in antiaromatic rings. Further substantiation of this conclusion is provided in section 5 of Supporting Information File 1.

For the N-containing heterocycles, we observe a similar dichotomy, although in this case all systems are aromatic. The two six-membered rings (pyridine and pyrazine) shift the distribution to the left and downwards of the baseline, towards lower HOMO and LUMO values (the effect is more pronounced for pyrazine than pyridine). Conversely, the five-membered ring (pyrrole) shifts it to the right and upwards, maintaining a similar HOMO range to COMPAS-1, but extending into much higher LUMO values. The behavior of the various N-containing rings is well documented in the literature [17,44–49], although to the best of our knowledge, these three systems have never been compared directly before in a data-driven manner.

These findings are summarized in a more quantitative manner in Figure 9B. In this set of stacked histogram plots, the colored rectangles represent the relative prevalence of the various building blocks (in this case, we also included cyclobutadiene as a non-benzene building block). These plots reiterate the findings described above for the HOMO and LUMO properties and provide further information regarding the change in prevalence for each building block across the other property spaces, as well. In the interest of conciseness, we provide a detailed MO-based rationalization for all of the trends in section 6 of Supporting Information File 1.

Having studied the property space covered by the individual heterocycles, we performed a final analysis to investigate the effects of having multiple building blocks of a certain type in a single cc-hPAS (regardless of the presence and number of the other building blocks). To circumvent the size dependency, we once again focused only on the 9-ring systems. For this subset of molecules, we plotted the various molecular properties as a function of the number of building blocks of each type (from 1–4; the number of examples containing more than 4 building blocks of a single type are too few to be statistically meaningful).

**Figure 10:**
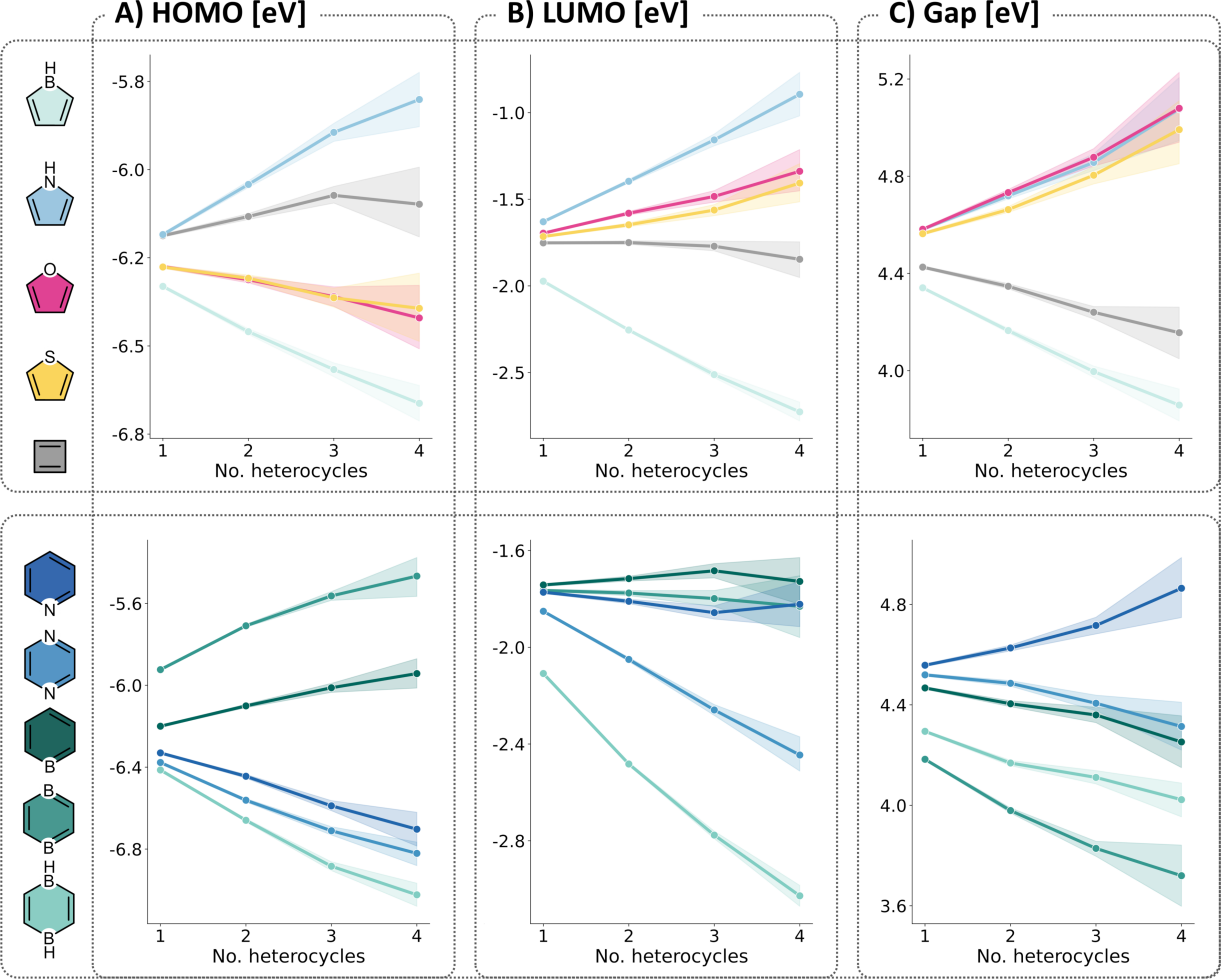
The effect of multiple heterocycles of a certain type on the A) HOMO, B) LUMO, and C) Gap. For all 9-ring systems in the dataset, we calculated the average property value of the rings containing 1, 2, 3, and 4 building blocks of a certain type, respectively. The data corresponding to each building block and each number of repeating units is represented by points corresponding to the color-coding shown on the left. For clarity, we have separated the 4- and 5-membered rings (top row) from the 6-membered rings (bottom row) and connected the points in each series by lines to assist in visual identification. The shading represents the 95% confidence interval of the value.

Not surprisingly, the trends shown in Figure 10 reiterate and further substantiate some of the previous findings, however they also reveal additional information. Primarily, these plots demonstrate the cumulative effect of incorporating multiple rings. Furthermore, the slopes of the lines indicate the strength of the effects – e.g., it can be seen clearly that pyrrole has a much stronger effect on the HOMO values than either furan or thiophene, and that 1,4-diborinine has the strongest LUMO-lowering effect of all building blocks in our library. In addition, these plots can provide further insight into the three building blocks that showed similar coverage as the cc-PBHs, namely, furan, thiophene, and cyclobutadiene. Although the distribution plot itself indicated a weak or negligible effect of these building blocks, Figure 10 reveals that indeed they do influence the molecular properties. Cyclobutadiene appears to have very little effect on the LUMO but does contribute to raising the HOMO and therefore decreasing the Gap. Furan and thiophene display very similar behaviors, as can be seen from the slopes of their plots for all three properties. An additional version of this figure that includes the benzene trend in provided in section 3.3 of Supporting Information File 1.

## Conclusion

We have performed a comprehensive data-driven analysis of the new dataset of *cata*-condensed hetero-polycyclic aromatic systems, COMPAS-2, which contains over 500,000 molecules. Following a comparison to cc-PBHs to establish a baseline for our study, our analysis was divided into three main levels, proceeding from low to high structural resolution: a) global properties, b) atomic composition, and c) building block composition. At each of these levels, we analyzed the data according to various structural features, to elucidate the underlying structure–property relationships and delineate clear principles that can aid in rational design of new cc-hPASs.

The main findings of our analysis are as follows:

1. Global features: this analysis revealed that molecular size affects electronic properties, but that the sensitivity to size becomes less apparent in larger molecules. Similarly, the overall electron count [(4*n*) or (4*n* + 2)] has a noticeable effect in smaller molecules but becomes unimportant in medium-sized and larger PASs. Finally, no specific trends were found between geometric features and molecular properties, except for the longest linear stretch. However, this effect is only clearly apparent in PASs that contain no antiaromatic rings.

2. Atomic composition: the analysis in this section revealed a clear “boron effect” (the presence of boron corresponds to high HOMO, low LUMO, small Gap). However, it could not be ascertained whether the boron effect stems solely from the presence of the atom, or from the fact that the boron atoms are often found in antiaromatic rings (borole, 1,4-dihydrodiborinine). Similarly, the N, O, and S atoms appeared more prevalent in molecules with high LUMOs and high HOMO–LUMO gaps, but it remained unclear whether this is due to the electronegativity of these atoms or their presence in aromatic building blocks.

3. Building block composition: the investigation in this section uncovered several findings. We observed that the molecular properties of cc-hPASs are dictated to some extent by the aromatic character of the building blocks contained in the molecule – the more antiaromatic rings there are, the lower the HOMO, LUMO, and Gap become. Further analysis revealed that, in fact, cyclobutadiene has a relatively small effect on the frontier molecule orbitals, thus the majority of observed trend stems from the B-containing antiaromatic rings. Indeed, we found that boron atoms have a strong impact on the molecular properties, however, the direction of this impact is in opposite directions, depending on whether the specific heterocycle is aromatic or antiaromatic. Furthermore, we observed an interesting divergence between pyrrole and the other five-membered aromatic rings. Although all three rings lead to an increase in the Gap, the pyrrole raises the HOMO and LUMO while furan and thiophene lower the HOMO and raise the LUMO. In addition, thiophene and furan show similar behavior both in the magnitudes of their effects and in the size of the property space they cover, whereas pyrrole displays a much stronger impact on the property values and a much more broadly distributed property space. This suggests that the properties of the pyrrole-containing cc-hPASs are much more sensitive to variations in structure than their furan- and thiophene-containing counterparts. The other N-containing building blocks, pyridine and pyrazine, lower both frontier molecular orbitals, with the pyrazine having a stronger effect, due to the fact that it contains two nitrogens. Indeed, we demonstrated that for all building blocks there is a cumulative effect on the properties, whereby incorporation of multiple building blocks continuously impacts the molecular properties.

To the best of our knowledge, this is the first data-driven investigation of this kind. It provides for the first time a clear overview of the property space that is achievable with these molecules, as well as detailed information on how to access different parts of this property space through structural design. The insight gleaned from this analysis not only deepens our understanding of the chemical properties of these important molecules, but also provides us with important tools for designing new molecules with desired properties. We emphasize that there is still much more to be learned from this rich database, including the reciprocal effects of adjacent building blocks, the importance of multi-ring substructures, and the interplay of different building blocks. Combining different types of heterocycles has been found to endow cc-hPASs with promising properties (e.g., pyrrole and thiophene [50], pyrrole and furan [51], borinine and thiophene [52]). Our data now shines new light on the interplay of these building blocks, but the exact relationships governing the resulting properties are unknown. Such complex relationships require more advanced data-analysis tools, and we are currently leveraging different machine learning and deep learning techniques to tap the full potential of the COMPAS-2 dataset.

## Supporting Information

The COMPAS-2 dataset is freely available online at the Poranne Group repository: https://gitlab.com/porannegroup/compas.

File 1 Further discussion and additional visualizations, an MO-based explanation of the chemical trends detailed in this analysis.

## Data Availability

The data used for/in this study is openly available in Figshare at https://doi.org/10.6084/m9.figshare.24347152 and on Gitlab at https://gitlab.com/porannegroup/compas. The data was derived from sources available in the public domain [COMPAS-2, 1/2024].
