# Supplementary material for: Hetero-polycyclic aromatic systems: A data-driven investigation of structure–property relationships
Source: Beilstein J Org Chem. 2024 Jul 31;20:1817–30. doi: 10.3762/bjoc.20.160 (PMC11301039; doi:10.3762/bjoc.20.160)
Supplement: File 1 — Further discussion and additional visualizations, an MO-based explanation of the chemical trends detailed in this analysis. [file Beilstein_J_Org_Chem-20-1817-s001.pdf]

BEILSTEIN JOURNAL OF ORGANIC CHEMISTRY

Supporting Information

for

Hetero-polycyclic aromatic systems: A data-driven investigation of structure–property relationships

Sabyasachi Chakraborty, Eduardo Mayo Yanes and Renana Gershoni-Poranne

Beilstein J. Org. Chem. **2024,** *20,* 1817–1830. doi:10.3762/bjoc.20.160

Further discussion and additional visualizations, an MO-based explanation of the chemical trends detailed in this analysis

License and Terms: This is a supporting information file under the terms of the Creative Commons Attribution License (https://creativecommons.org/licenses/by/4.0). Please note that the reuse, redistribution and reproduction in particular requires that the author(s) and source are credited and that individual graphics may be subject to special legal provisions.
The license is subject to the *Beilstein Journal of Organic Chemistry* terms and conditions: (https://www.beilstein-journals.org/bjoc/terms)

Table of contents

1. Additional global features analysis S2
  1.1. Effect of longest linear stretch S2
  1.2. Effect of non-planarity S3
  1.3. Effect of number of branches S4
  1.4. Effect of 4*n* versus 4*n* + 2 π-electron count S5
2. Effect of atomic composition S7
3. Effect of heterocycle composition S8
  3.1. Size of ring S8
  3.2. Effect of number of antiaromatic rings S9
  3.3. Effect of number of heterocycles S11
4. The coverage of property space S12
5. The effect of boron atoms S14
6. Molecular orbitals-based rationalization of heterocyclic trends S16
  6.1. Aromatic six-membered rings S16
  6.2. Aromatic five-membered rings S18
  6.3. Antiaromatic boron-containing rings S22
7. References S25

1. Additional global features analysis

In the main text, we mention that additional global features were studied, namely: deviation from planarity, prevalence of angular annulation, and the number of branching points. These analyses are presented in this section.

1.1. Effect of longest linear stretch

In the main text, we describe the stratification of the property space of COMPAS-1 according to the longest linear stretch. To clarify this point, we include here the scatter plot of the HOMO/LUMO property space for COMPAS-1 and COMPAS-2, colored according to the number of rings in the longest linear stretch (**Figure S1**). The plots show a clear trend (more pronounced for COMPAS-1), whereby longer stretches lead to lower LUMOs. The HOMOs do not show a clear trend, although there does seem to be a narrowing of the range with longer linear stretches.

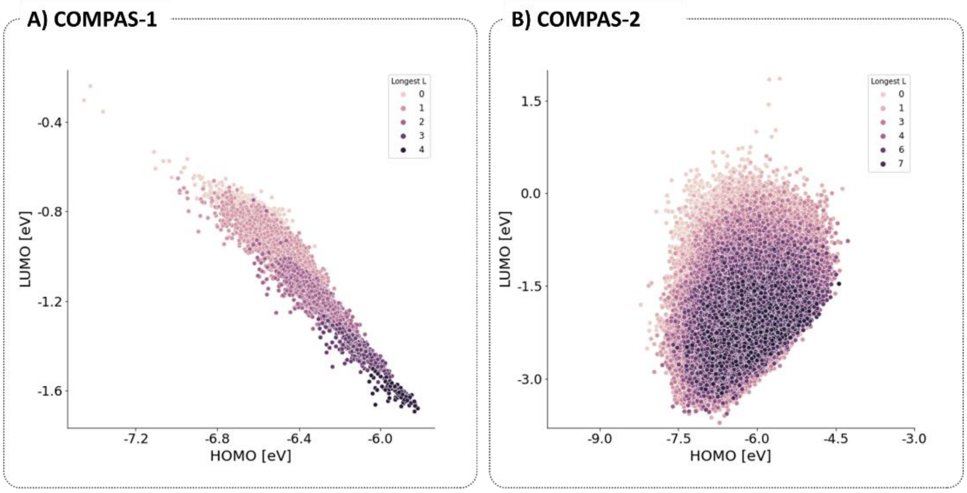

Figure S1: *Scatter plot of the HOMO/LUMO property space for A) COMPAS-1 and B) COMPAS-2, colored according to the number of rings in the longest linear stretch.*

1.2. Effect of non-planarity

To investigate the effect of non-planarity of the molecules, we calculated the deviation from planarity for each molecule in the dataset. This was achieved by placing the molecule in the *xy* plane and calculating the maximal distance between two atoms on the *z* axis, denoted as Δz. Figure S2 displays the distribution of molecules along the HOMO/LUMO and AEA/AIP property spaces, with each data point colored according to its Δz. Only molecules with $\Delta z > 1\text{Å}$ are plotted. As can be seen from the plots, there is no discernible trend or tendency.

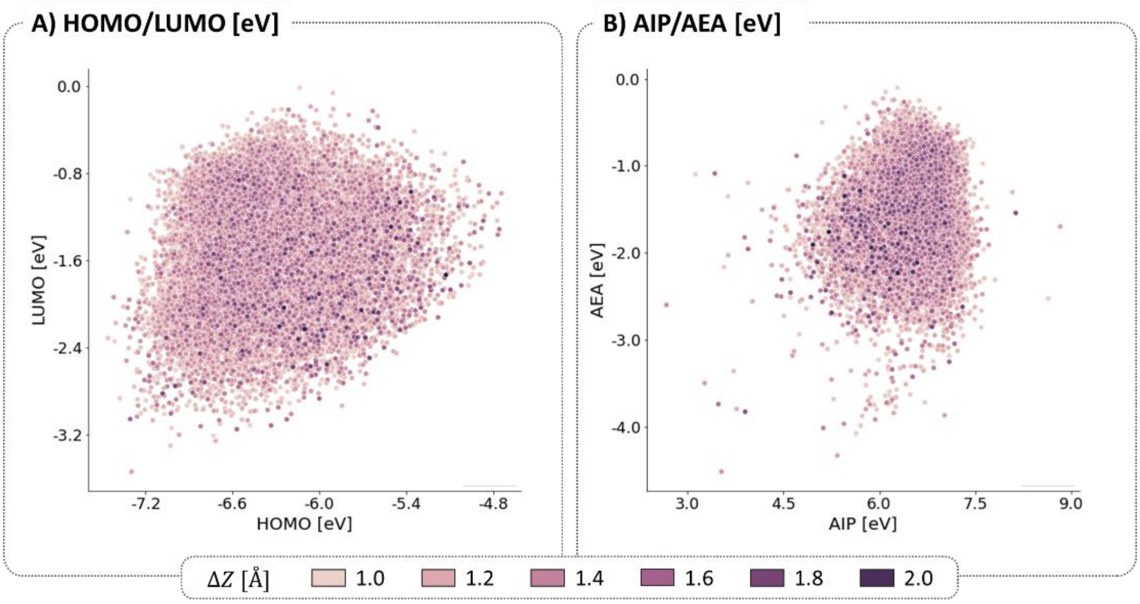

Figure S2: *Scatter plots of A) HOMO/LUMO and B) AIP/AEA property spaces, colored by the deviation from planarity of the molecules (as measured by* Δz*).*

1.3. Effect of number of branches

To investigate whether the number of branching points has an effect on any of the molecular properties studied in this report, we plotted KDE plots of the various properties, colored by the number of branches (**Figure S3**). As can be seen from the plots, there is no discernible trend.

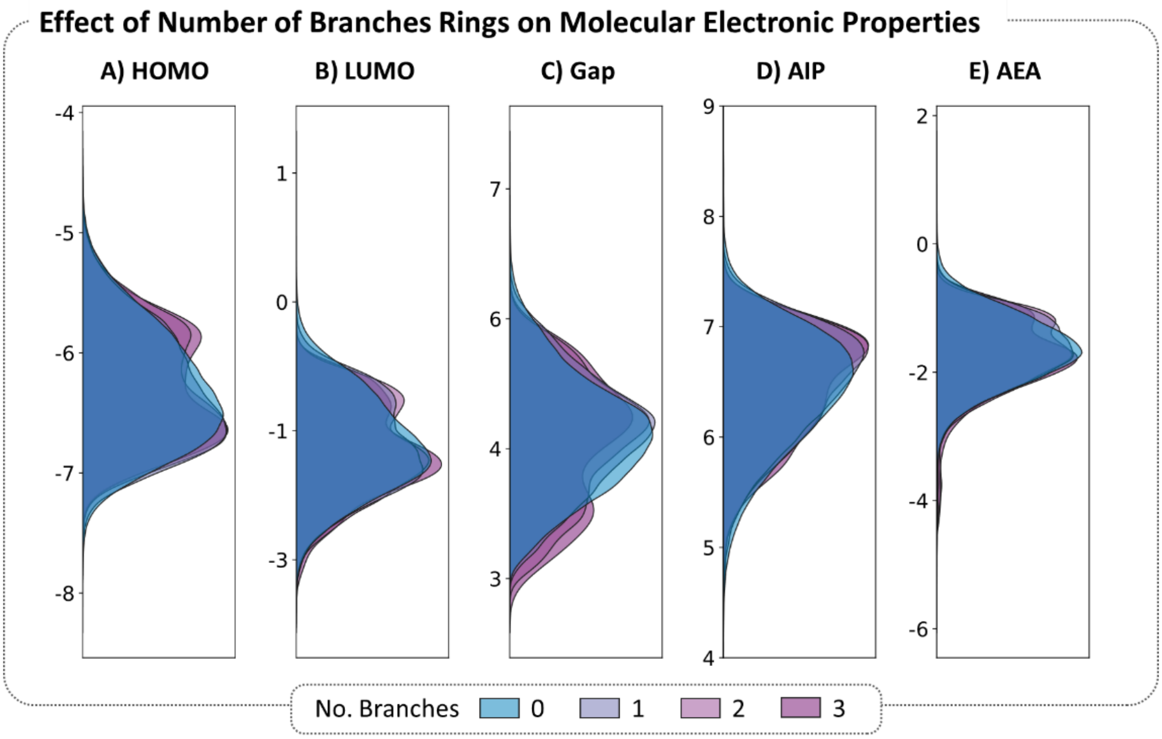

Figure S3: *KDE plots colored by number of branching points for the five molecular properties: A) HOMO, B) LUMO, C) Gap, D) AIP, and E) AEA.*

1.4. Effect of 4*n* versus 4*n* + 2 π-electron count

In addition to looking at the HOMO, LUMO and Gap ranges of the formally aromatic and formally antiaromatic cc-hPASs (shown in the main text), we also plotted the atomization energy of the (4*n*) and (4*n* + 2) subsets, divided by the number of rings (**Figure S4**). The atomization energy is a measure of the stability of the molecule. The plot shows that the (4*n* + 2) molecules consistently have higher atomization energies, indicating increased stability, at every size.

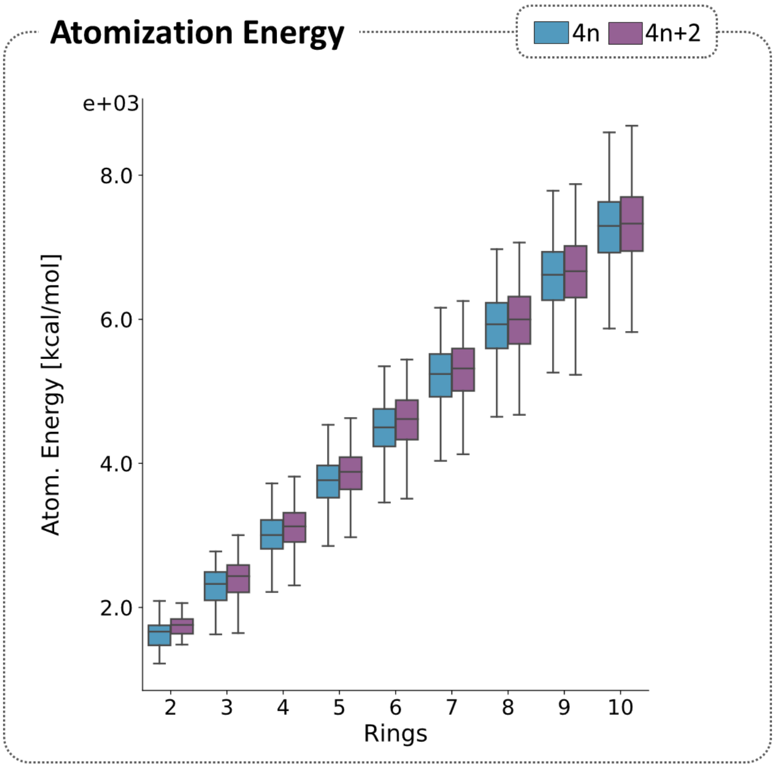

Figure S4: *The atomization energy for the (4n + 2) and (4n) molecules, grouped by number of rings in the molecule.*

To further investigate whether the formal aromatic character of the molecule (i.e., global π-electron count) has an effect on any of the molecular properties studied in this report, we divided the dataset into two subsets: molecules with (4*n*) π-electrons (total of 246,051 molecules) and molecules with (4*n* +2) π-electrons (total of 278,336 molecules). We then plotted KDE plots of the various properties for each of these subsets, colored by the number of rings (**Figure S5**). As can be seen from the plots, there is no discernible difference between the two subsets, in terms of the relationship between the molecular properties and number of rings.

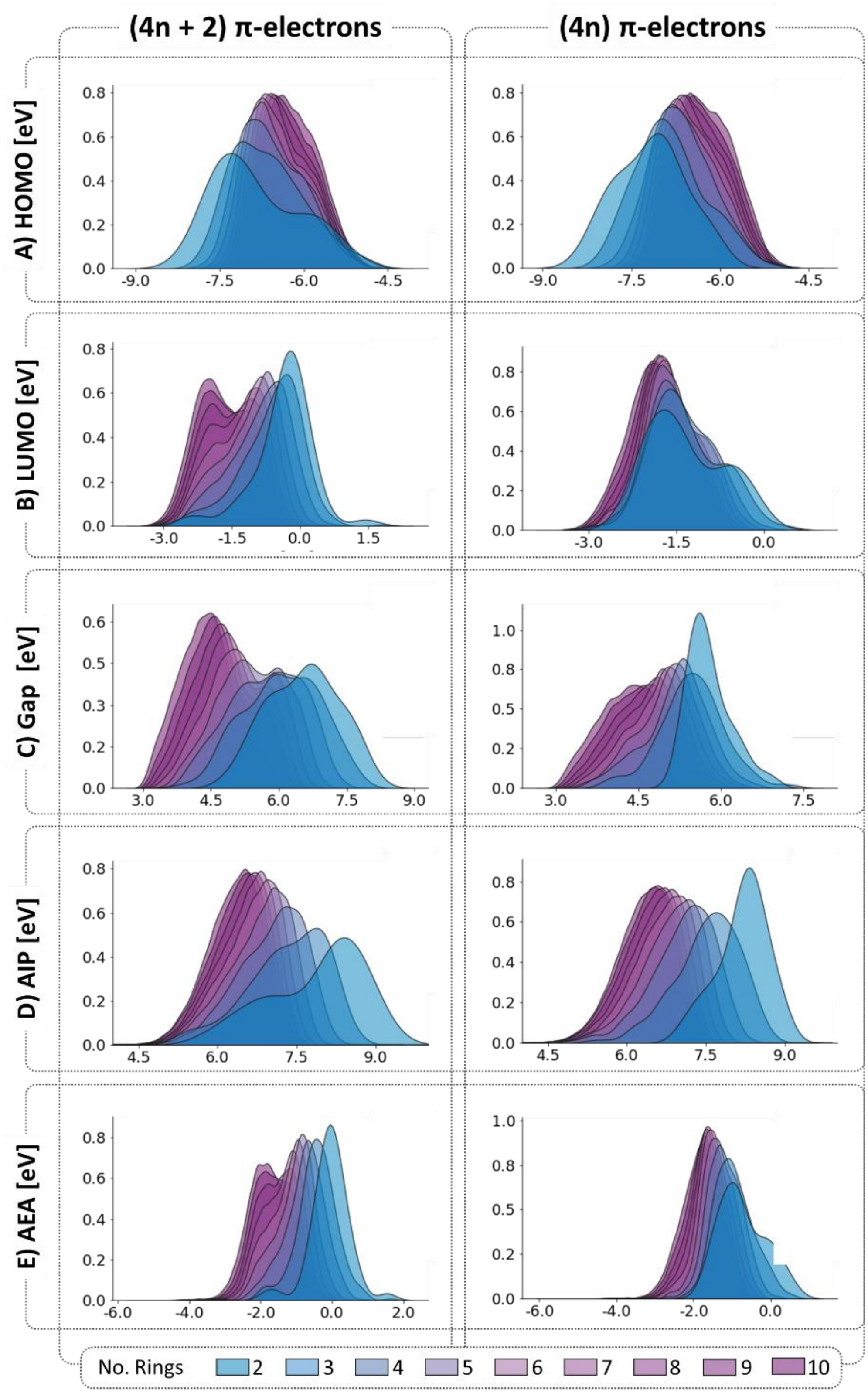

Figure S5: *KDE plots, colored by number of rings, plotted separately for the (4n + 2) and (4n) molecules (left and right, respectively) for the five molecular properties: A) HOMO, B) LUMO, C) Gap, D) AIP, and E) AEA.*

2. Effect of atomic composition

We visualized the distribution of the various types of heteroatoms across the property space by generating a series of scatter plots (HOMO/LUMO and AIP/AEA) and coloring each plot according to the number of heteroatoms of a certain type.

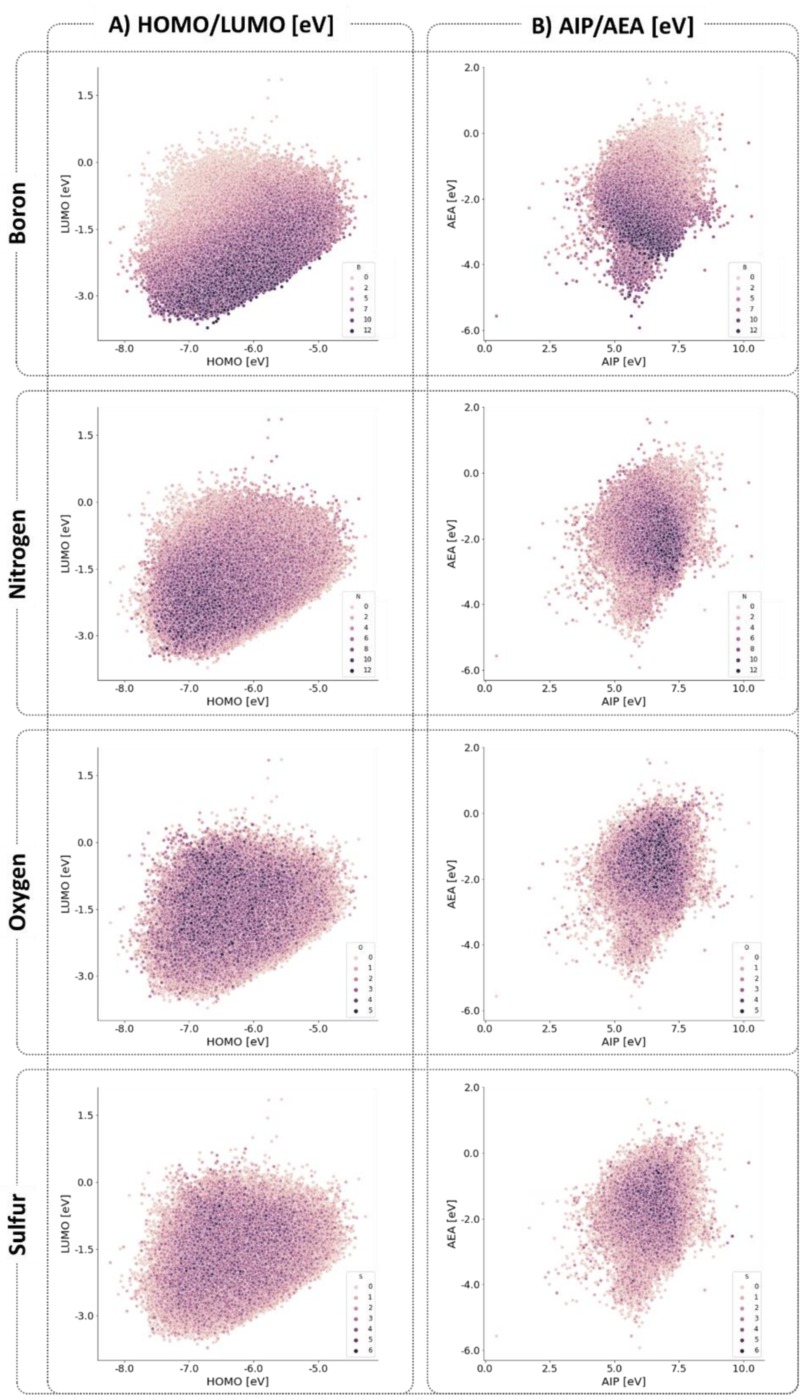

Figure S6: *Scatter plots of the A) HOMO/LUMO and B) AIP/AEA property spaces, colored by the different heteroatoms.*

3. Effect of heterocycle composition

3.1. Size of ring

As mentioned in the main text, the first descriptor we used to differentiate between the various building blocks was their size: 4-, 5-, or 6-membered rings (4MRs, 5MRs, 6MRs, respectively). To do this, we plotted KDE distributions of the various properties, colored by the number of rings of the different sizes (**Figure S7**). To avoid the size-dependency, the plots only consider the 9-ring systems.

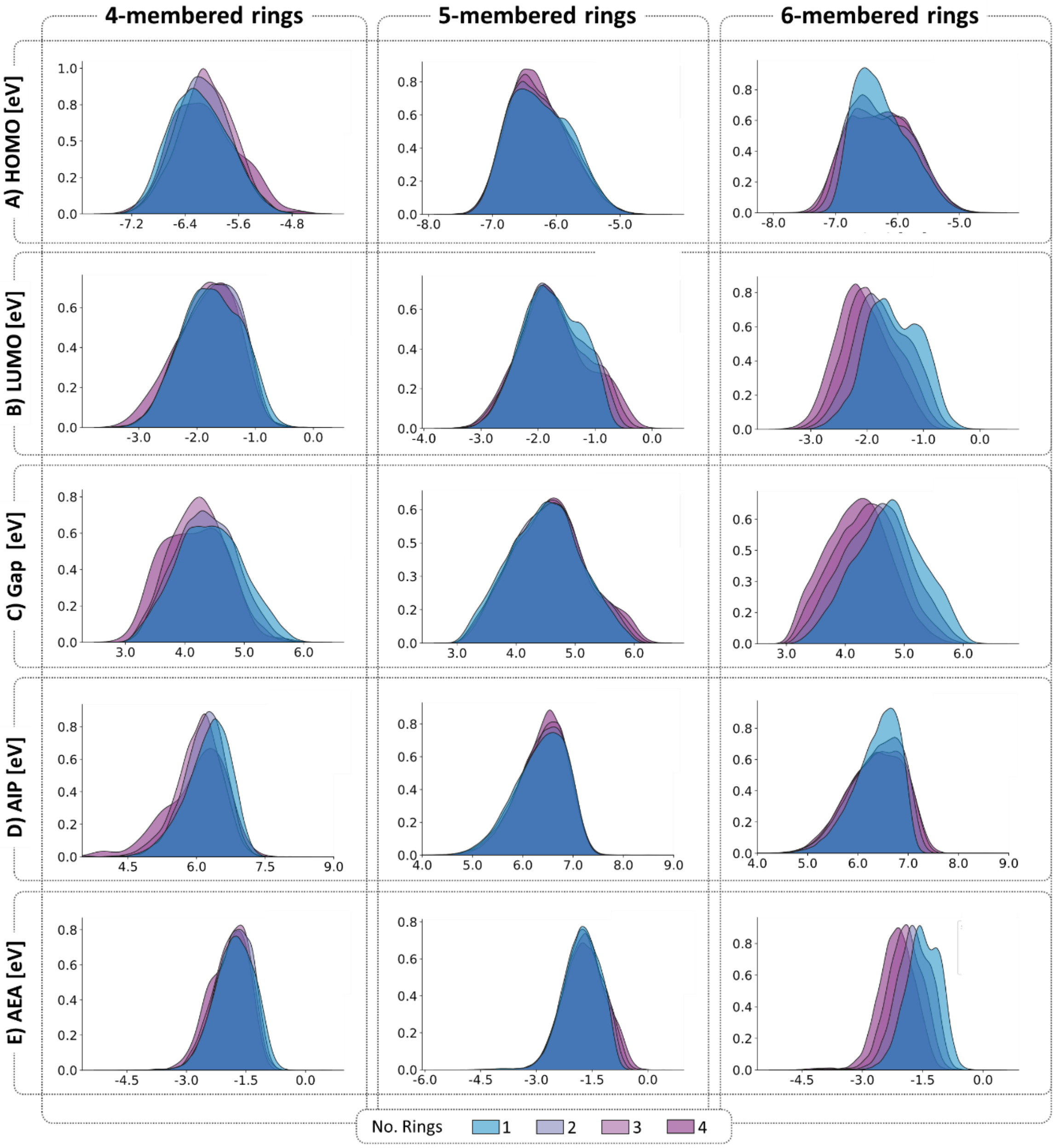

Figure S7: KDE distributions colored by the number of rings of a certain size for the five molecular properties: A) HOMO, B) LUMO, C) Gap, D) AIP, and E) AEA. The columns show the effect of increasing the number of 4MRs (left), 5MRs (center), and 6MRs excluding benzene (right).

The plots indicate that for both 4MRs and 5MRs, there is no discernible relationship between increasing the number of rings of this size and changing the molecular properties. For the 6-membered rings, a drift can be seen for the LUMO, Gap, and AEA. Most likely, the effect is primarily a lowering of the LUMO, which is then reflected also in a decrease in the Gap and in

the AEA (according to Koopmans' theorem, the LUMO provides a good approximation for electron affinity). We hypothesize that this behavior is a result of the 1,4-dihydrodiborinine—an antiaromatic 6MR building block that has the most substantial LUMO lowering effect. As the number of 6MRs is increased, the likelihood increases of one or more of them being 1,4-dihydrodiborinine.

3.2. Effect of number of antiaromatic rings

In addition, we studied the effect of the number of antiaromatic rings on the molecular properties, for each of the two subsets. As can be seen in **Figure S8**A, the two subsets show a similar trend: increasing the number of antiaromatic moieties leads to a drop in LUMO values as well as a shift towards lower HOMO values, regardless of the overall π-electron count of the molecule. Similarly, both show a localization of molecules with a high number of antiaromatic rings for the AIP/AEA plot (**Figure S8**B).

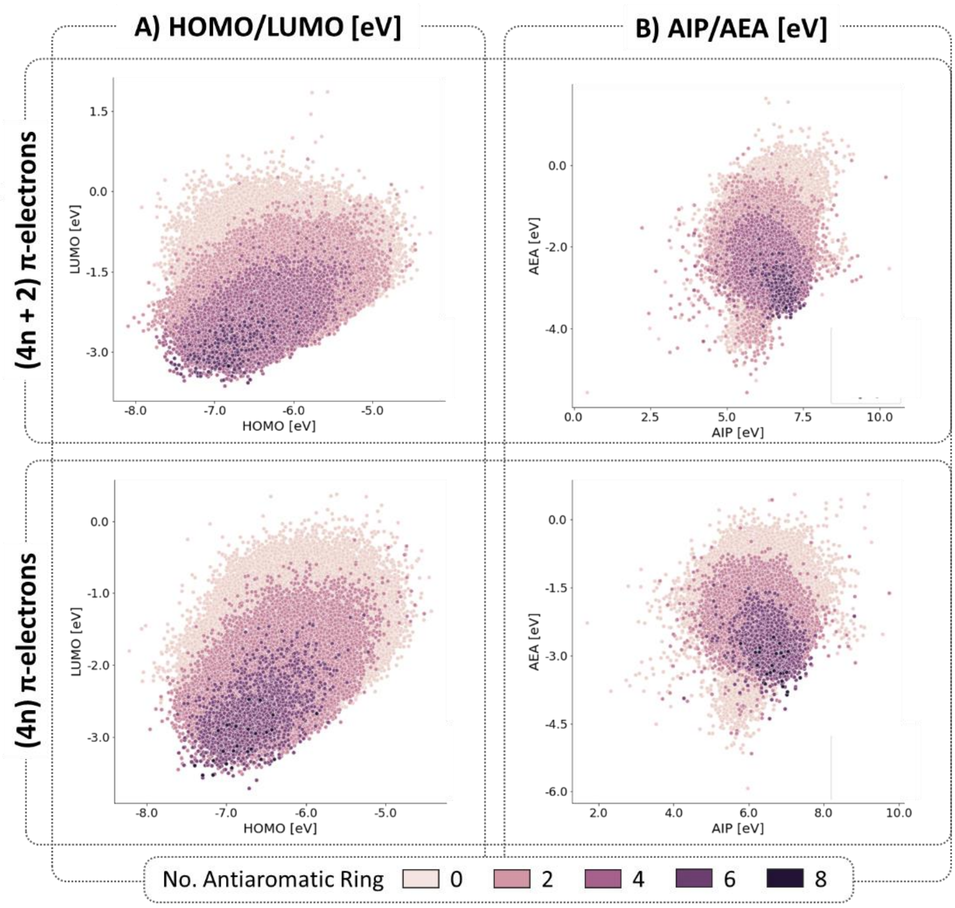

Figure S8: *Scatter plots of A) HOMO/LUMO and B) AIP/AEA property spaces for the 4n + 2 (top) and 4n (bottom) subsets, colored by the number of antiaromatic rings.*

In contrast, a certain trend can be observed when coloring the same data distribution according to the longest linear stretch (**Figure S9**). For the formally Hückel aromatic molecules, molecules with longer stretches appear in lower LUMO values and higher HOMO values. The highest LUMO values are dominated by molecules with no linear stretch, or a very short one.

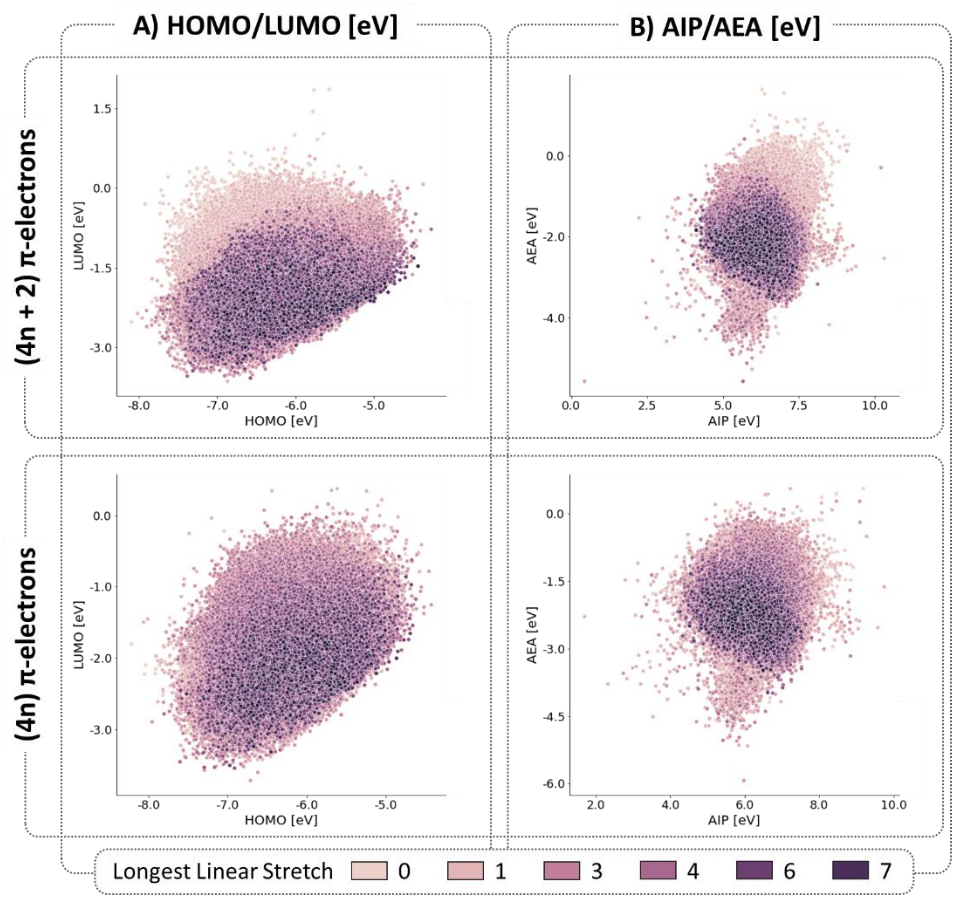

Figure S9: *Scatter plots of A) HOMO/LUMO and B) AIP/AEA property spaces for the 4n + 2 (top) and 4n (bottom) subsets, colored by the longest linear stretch.*

3.3. Effect of number of heterocycles

In Figure 10 of the main text, we show the various molecular properties as a function of the number of building blocks of each type (from 1–4; the number of examples containing more than 4 building blocks of a single type are too few to be statistically meaningful). As we mentioned in the text, these plots demonstrate the cumulative effect of incorporating multiple rings. To reiterate this point, we show below (**Figure S10**) another version of the same figure, this time including the line for the benzene rings (light gray).

As seen in the bottom row of **Figure S10**, the benzene trend is opposite to the average of the heterocycle trends. This is because the molecules all have the same total number of rings (*n* = 9), and therefore an increase in the number of benzenes comes at the expense of any type of heterocycle.

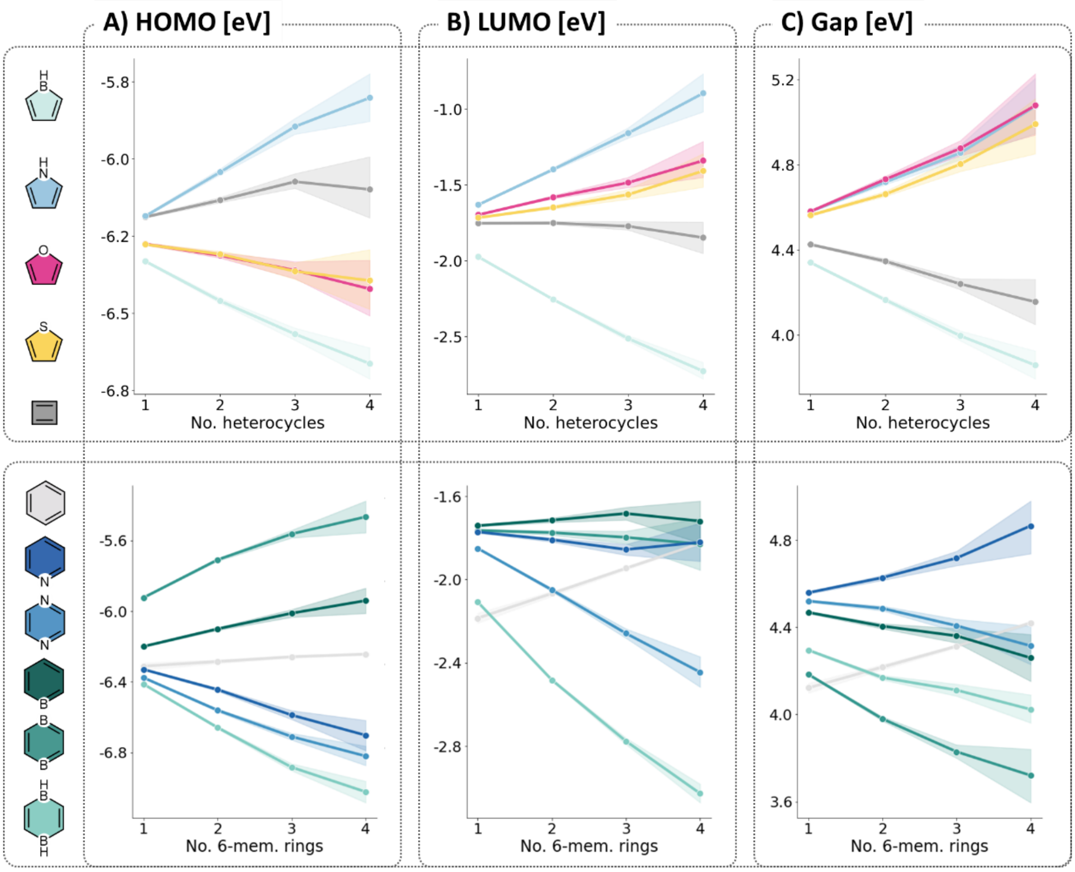

Figure S10: *The effect of multiple heterocycles of a certain type on the A) HOMO, B) LUMO, C) Gap. For all 9-ring systems in the dataset, we calculated the average property value of the rings containing 1, 2, 3, and 4 building blocks of a certain type, respectively. The data corresponding to each building block and each number of repeating units is represented by points corresponding to the color-coding shown on the left. For clarity, we have separated the 4- and 5-membered rings (top row) from the 6-membered rings (bottom row) and connected the points in each series by lines to assist in visual identification. The shading represents the 95% confidence interval of the value.*

4. The coverage of property space

In the main text, we show how incorporation of each type of heterocycle affects the coverage of the HOMO/LUMO property space. To complement this, in **Figure S11** we show the combined coverage of all N-and B-containing heterocycles, respectively.

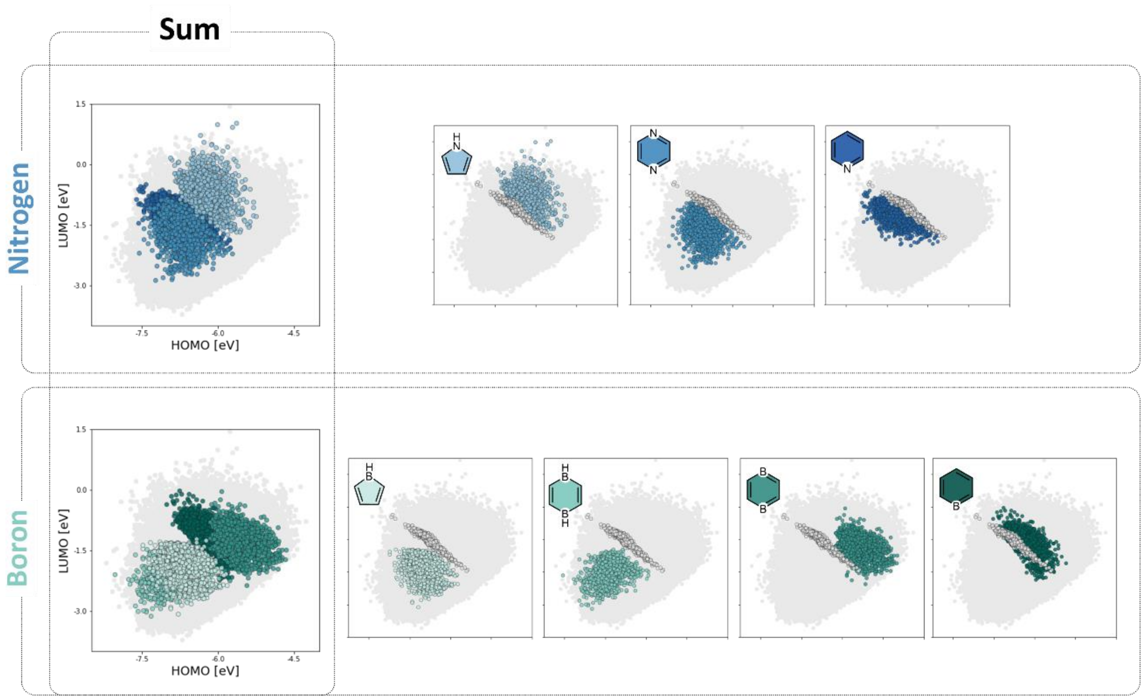

Figure S11: *Scatter plots of the HOMO/LUMO property space, colored according to the various N-containing (top) and B-containing (bottom) heterocycles. The combined coverage of the respective groups is shown in the Sum column.*

By looking at the individual regions covered and at the sum of the coverage of the N- and B-containing rings, one can observe that there are certain regions that are never accessed by a single type of heterocycle/heteroatom, yet they appear in the background as part of the COMPAS-2 property space. This indicates that these regions are accessed by the combination of different heterocycles/heteroatoms. To demonstrate this, we plotted the same scatter plots, this time coloring the molecules that contain a combination of two heterocycles (**Figure S12**). The shifting of the covered region suggests a cumulative effect of the different building blocks (rather than an unpredictable, emergent effect). In addition, for pairs of building blocks with contradicting effects, it allows us to discern which building block has a stronger effect.

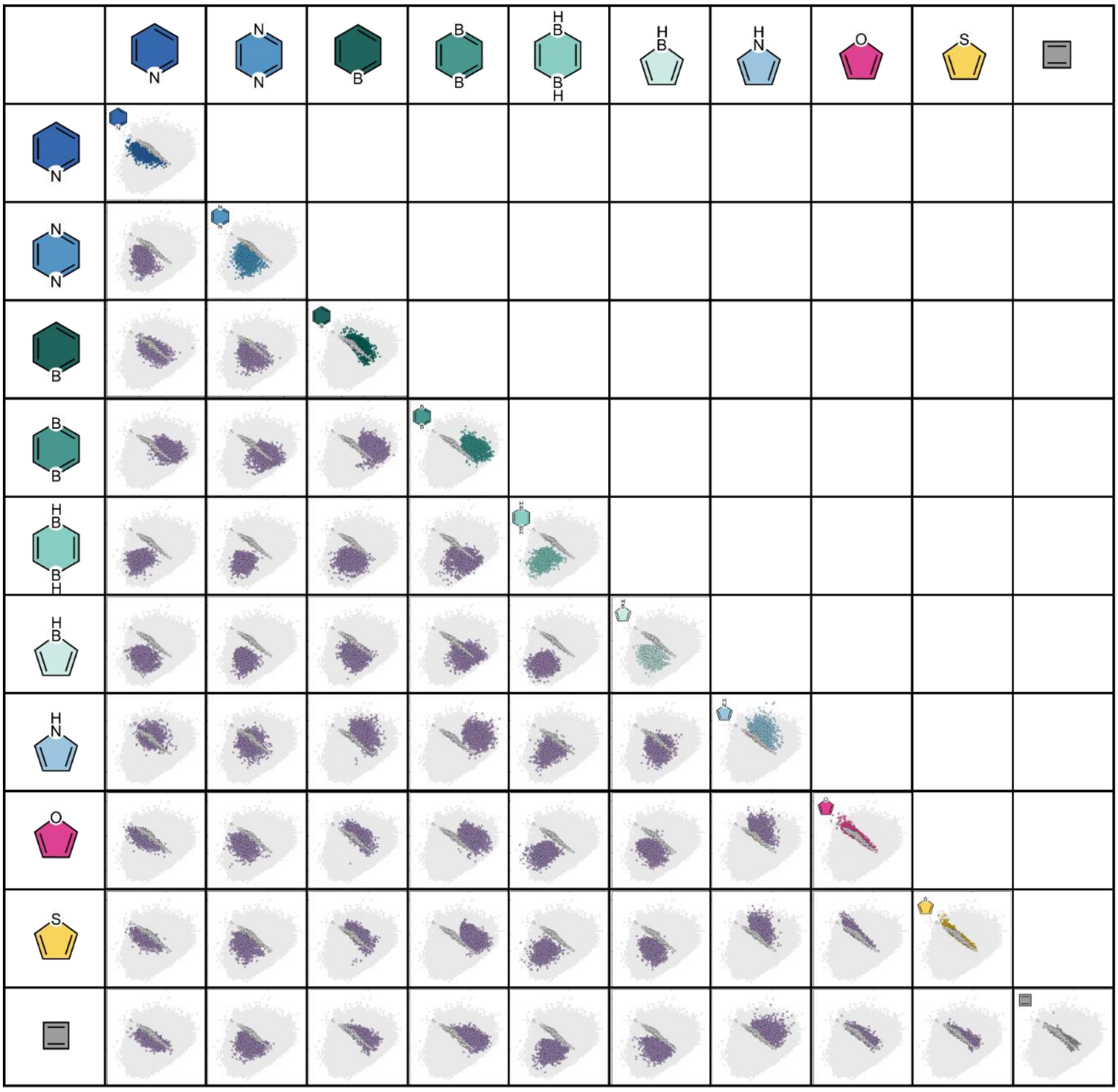

Figure S12:*Scatter plots of the HOMO/LUMO property space, colored by different binary combinations of building blocks (colored in purple). In each plot, the reference of cc-PBHs is shown in gray.*

5. The effect of boron atoms

In the main text, we present and discuss the contrasting effects of boron atoms in aromatic versus antiaromatic rings. In this section, we provide additional analyses that support the conclusions presented in the main text.

In **Figure S13**, we show the HOMO/LUMO property space, colored by the number of boron atoms of a certain type (aromatic or antiaromatic). The plots clearly show the tendency of the B atoms to accumulate in different regions, depending on the aromatic character of the heterocycle they belong to.

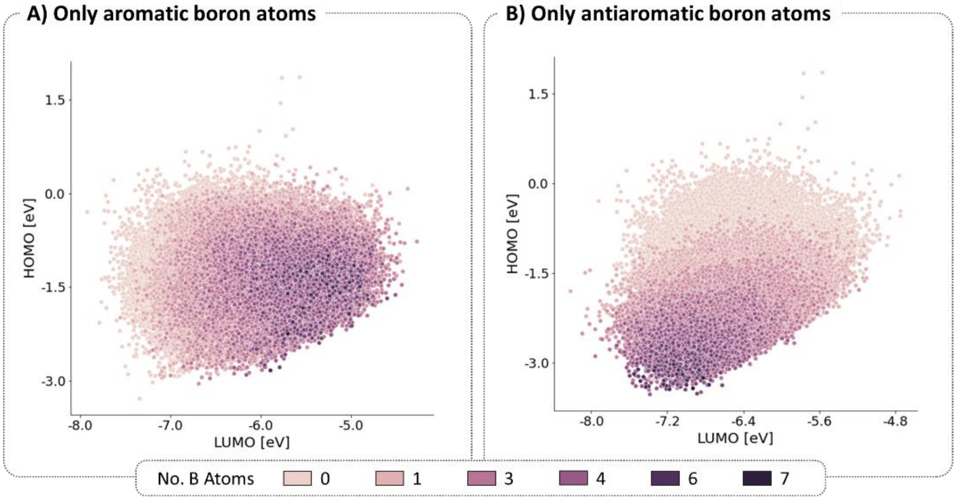

Figure S13: *Scatter plots of the HOMO/LUMO property space, colored according to the number of B atoms, separated into A) only aromatic B atoms (molecules with antiaromatic Bs not shown) and B) only antiaromatic B atoms (molecules with aromatic Bs not shown).*

In **Figure S14** we show the same property spaces, but this time we show only 9-ring systems (to circumvent the size dependency) and only those that contain either 4 (top) or 3 (bottom) B-containing rings. In other words, each of the points on each plot contains the same number of total rings and the same number of B-containing rings. The individual data points are colored according to the number of rings of a certain type: the aromatic B-containing rings are colored in a blue gradient and the antiaromatic B-containing rings are colored in a red gradient. The plots demonstrate very clearly that the two types of rings have different impacts on the molecular properties. While the antiaromatic B-containing rings shift the HOMO and LUMO to lower values, the aromatic B-containing rings shift the HOMO and LUMO to higher values.

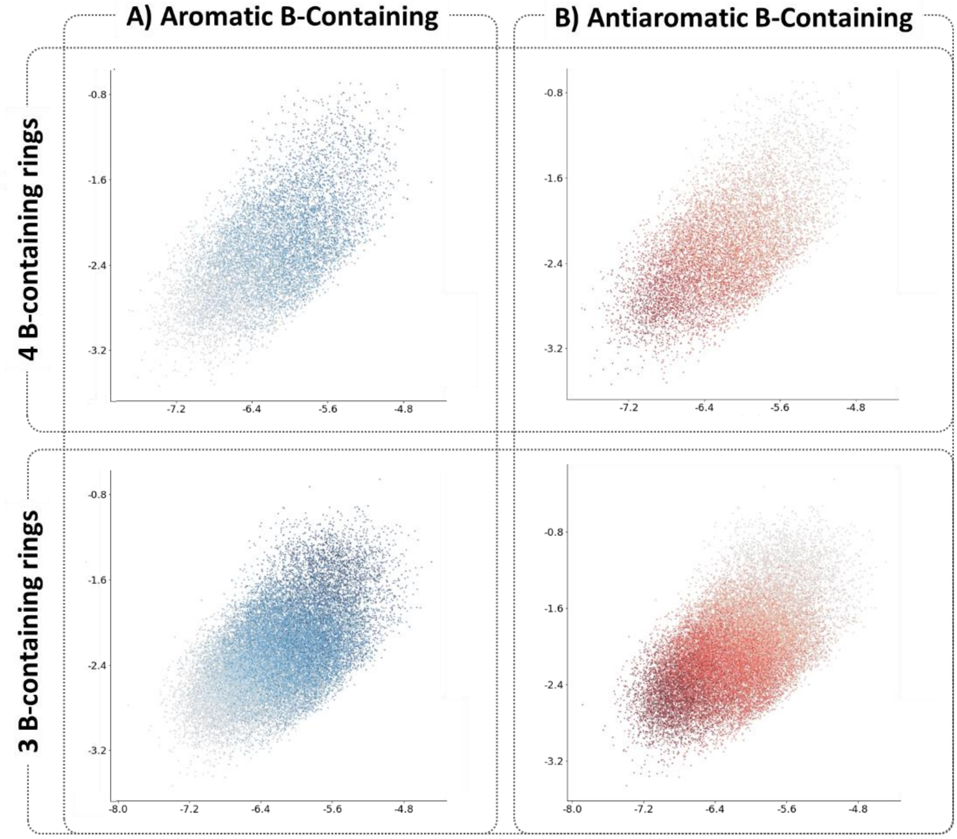

__Figure S14:__ Scatter plots of the HOMO/LUMO (x/y) property space colored by the number of A) aromatic B-containing rings and B) Antiaromatic B-containing rings. Top row: 9-ring systems containing 4 B-containing rings; Bottom: 9-ring systems containing 3 B-containing rings.

6. Molecular orbitals-based rationalization of heterocyclic trends

In this section, we provide further explanation of the trends we observed for the various building blocks. In our analysis, we separate between the five- and six-membered building blocks, as their electronic structure is fundamentally different. To demonstrate the effects of the various building blocks, we study their benzannulated derivatives and derive conclusions regarding the participation of the heteroatom in the frontier MOs of the cc-hPAS.

6.1. Aromatic six-membered rings

There are two aromatic B-containing and two aromatic N-containing 6MR building blocks in our library.

For all four of these systems, we compare the frontier MOs with those of the analogous cc-PBH, naphthalene (Figure S14). In all the cases, we note that the HOMOs of the molecules are π-orbitals, and that they are very similar to the HOMO of naphthalene (with the exception of benzoborinine that has the B in the position non-adjacent to the fused bond). This means that for all these molecules, the *p* orbital of the heteroatom is participating in the HOMO, and the lone pair (for N) or empty orbital (for B) is perpendicular to the π-system, lying in the molecular plane. Therefore, the effect of the heteroatoms on the HOMO is via their electronegativity. N is more electronegative than C, and therefore lowers the energy of the HOMO. B is less electronegative and therefore raises the HOMO. These expectations based on the MO depiction are in line with the data-driven conclusions we draw in the main text: pyridine- and pyrazine-containing cc-hPASs have lower HOMO values whereas borinine- and 1,4-diborinine-containing cc-hPASs have higher HOMO values.

The situation changes with the LUMO. The LUMO of the N-containing systems is also a π-orbital, meaning that the effect of the N is similar: it lowers the energy of the LUMO thanks to its higher electronegativity. This expectation is corroborated by our data analysis, which demonstrated that cc-hPASs containing pyridine and pyrazine have lower LUMOs than the analogous cc-PBHs, and that the lowering is more pronounced for the pyrazine (which contains two nitrogen atoms).

In contrast, the LUMO of the B-containing systems is the empty sp^2 orbital (or combination of two sp^2 orbitals) of the boron atom(s) in the molecule – i.e. a σ-orbital. Based on this, one would predict that further extending the conjugation of the system will not have a substantial effect on the energy of the LUMO. This is in full agreement with our data-driven conclusions, which show that the LUMO level of the systems containing borinine and 1,4-diborinine are in a similar range as the cc-PBHs.

As shown by Houk and co-workers, in some specific cases of PASs with high symmetry and multiple N-heterocyclic rings, the HOMO may be a combination of the in-plane N lone pairs.[1] However, in the majority of cases, the frontier MOs are π-orbitals and their energies are lowered by the higher electronegativity of the nitrogen atoms (a similar conclusion to ours). It has been shown that the effect is cumulative, meaning that the impact on the MO energies increases with the addition of more nitrogen atoms. Furthermore, the properties of such systems are sensitive to the exact position of the pyridine/pyrazine ring within the PAS,[2] as well as to the exact position of the nitrogen (e.g., adjacent to the fused bond or non-adjacent). This explains the substantial broadening of the property space that we observe. It should be noted that incorporating such heterocycles into polycyclic systems is a well-known strategy for lowering both HOMO and LUMO values and is commonly utilized in the design of organic electronics.[1–5]

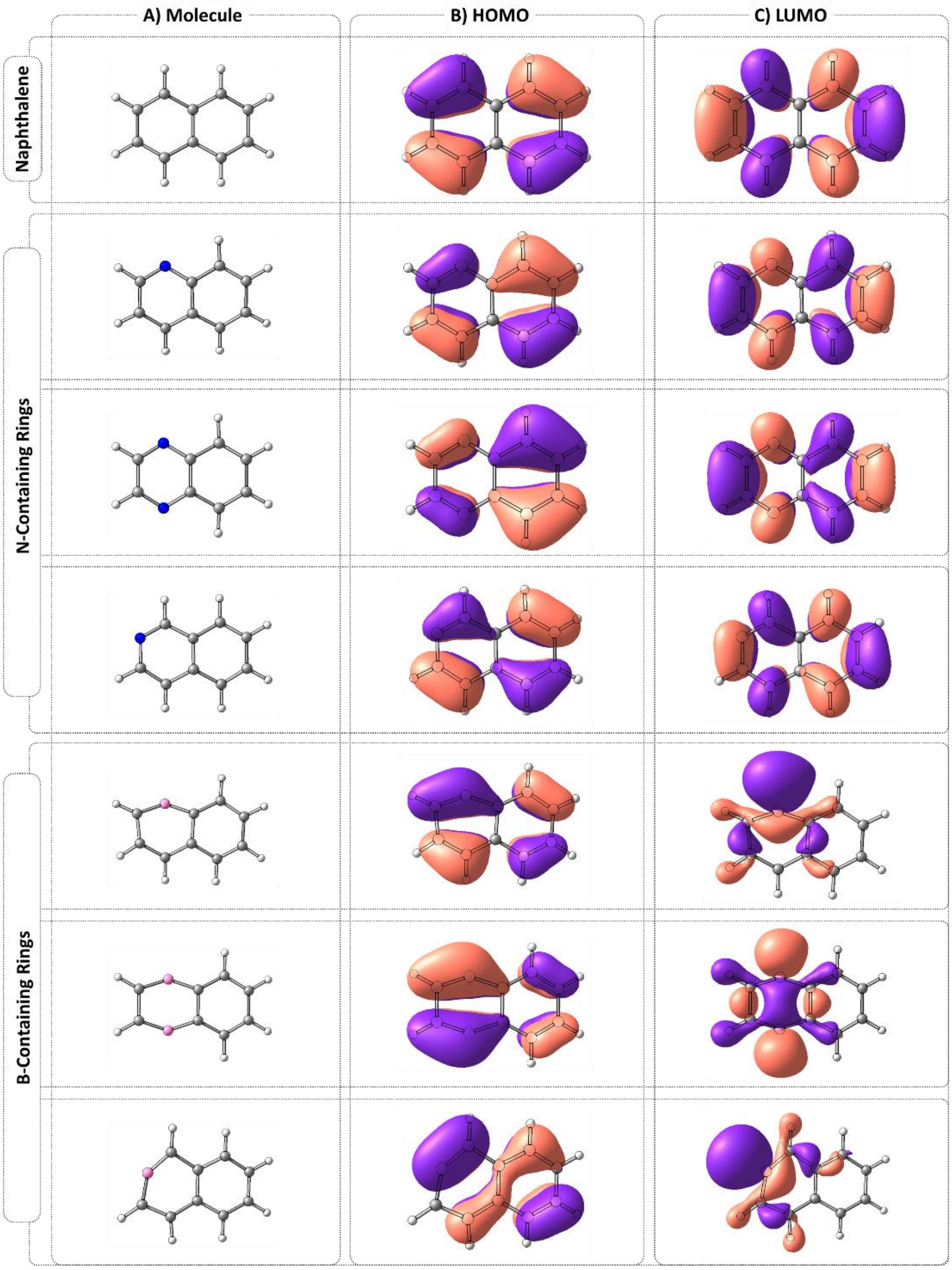

Figure S15: *Frontier molecular orbitals of naphthalene and benzannulated derivatives of pyridine, pyrazine, borinine, and 1,4-diborinine.*

6.2. Aromatic five-membered rings

For the aromatic 5MRs in our library (furan, pyrrole, and thiophene) the situation is somewhat different from the aromatic 6MRs. Here as well, incorporation can occur in one of two ways: either the heteroatom is positioned adjacent to the fused bond, or the heteroatom is positioned non-adjacent to the fused bond (Type A and Type B, respectively, **Figure S16**). However, in contrast to the 6MRs, the position of the heteroatom in this case has a much more substantial effect on the electronic structure – and therefore properties – of the molecule.

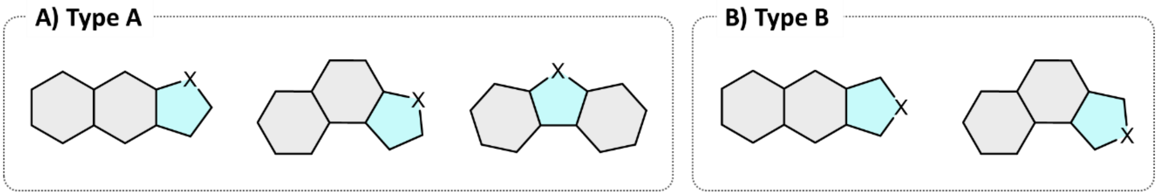

Figure S16: *Types of annulation of 5MR heterocycles: A) Type A – heteroatom adjacent to fused bond, B) Type B – heteroatom not adjacent to fused bond.*

For the Type A case, as noted by Momicchioli and Rastelli, "the chemical and physical properties of the benzoderivatives of the 5MR heterocycles should be interpreted on the basis of the properties of both benzene derivatives and naphthalene; one should speak of a series where the nature of the principal heteroatom gives rise to properties intermediate between those of the two extreme models."[6] In other words, one should consider such benzannulated derivatives on a spectrum between substituted benzene and naphthalene. The exact nature of the compound – i.e. where it lands on the spectrum – depends on the nature of the heteroatom.

Our results are in alignment with this insightful comment, both in terms of the data distributions and in terms of the MO analysis (**Figure S17**–**Figure S19**). On one end of the spectrum are the furan derivatives for which, as Momicchioli and Rastelli claim, "everything is as if the *p* orbitals of the heterocycles belonged to two separated substituents, $-CH=CH_2$ and −OH, and the properties of the molecule can be described by analogy with styrene". As shown in **Figure S17**, the HOMO and LUMO of benzofuran look like a superposition of the respective HOMOs and LUMOs of benzene and furan. Similarly, the HOMO and LUMO of dibenzofuran (**Figure S19**) appear like two separated benzene rings – as though the oxygen is a non-aromatic separator. This is in agreement with the data distribution of the furan-containing cc-hPASs shown in the main text. For these molecules, the ranges of property values are relatively similar to the cc-PBHs, with the exception that the LUMO values are slightly raised.

At the other end of the spectrum are the thiophene derivatives. The HOMO and LUMO of benzothiophene (**Figure S17**), naphthothiophene (**Figure S18**), and dibenzothiophene (**Figure S19**) look very similar to the parent PBH molecules, naphthalene and anthracene, respectively. This implies that the molecules' properties should also be quite similar to these molecules, with the exception that the sulfur may modulate the energy levels slightly. Indeed, the data distribution is exceedingly similar to the cc-PBHs (as shown in Figure 9 in the main text). The pyrrole moiety resides in the middle of this spectrum. For pyrrole, the π-system is distributed much more uniformly on the 5MR (seen for indole, **Figure S17**), which could explain its stronger impact on the molecular properties.

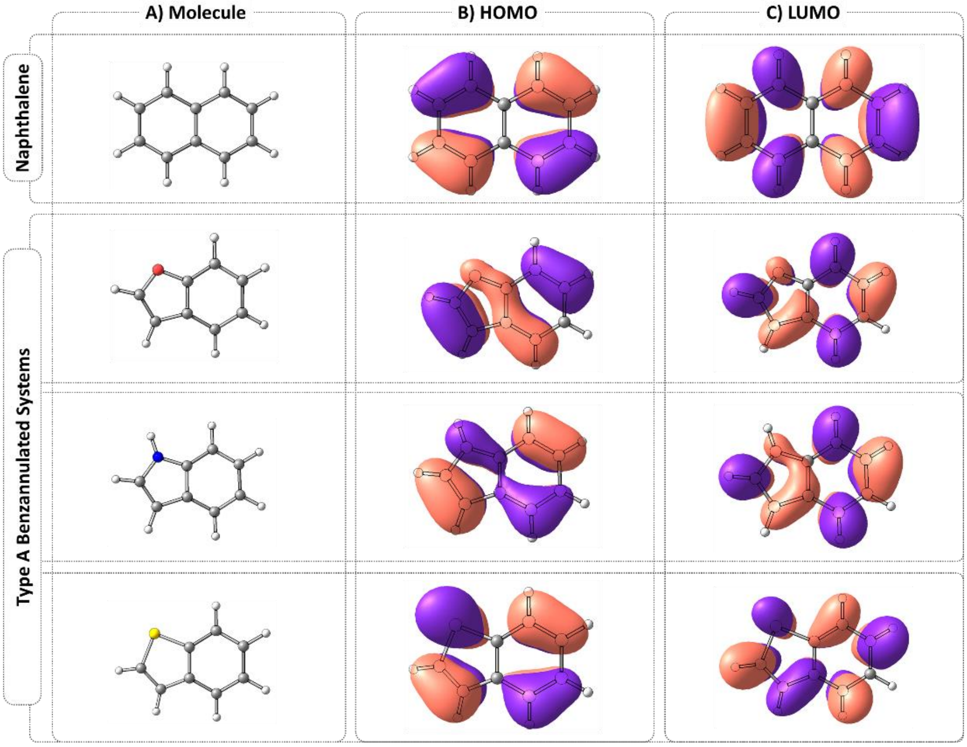

Figure S17: *Frontier molecular orbitals of naphthalene and Type A bicyclic derivatives of furan, pyrrole, and thiophene.*

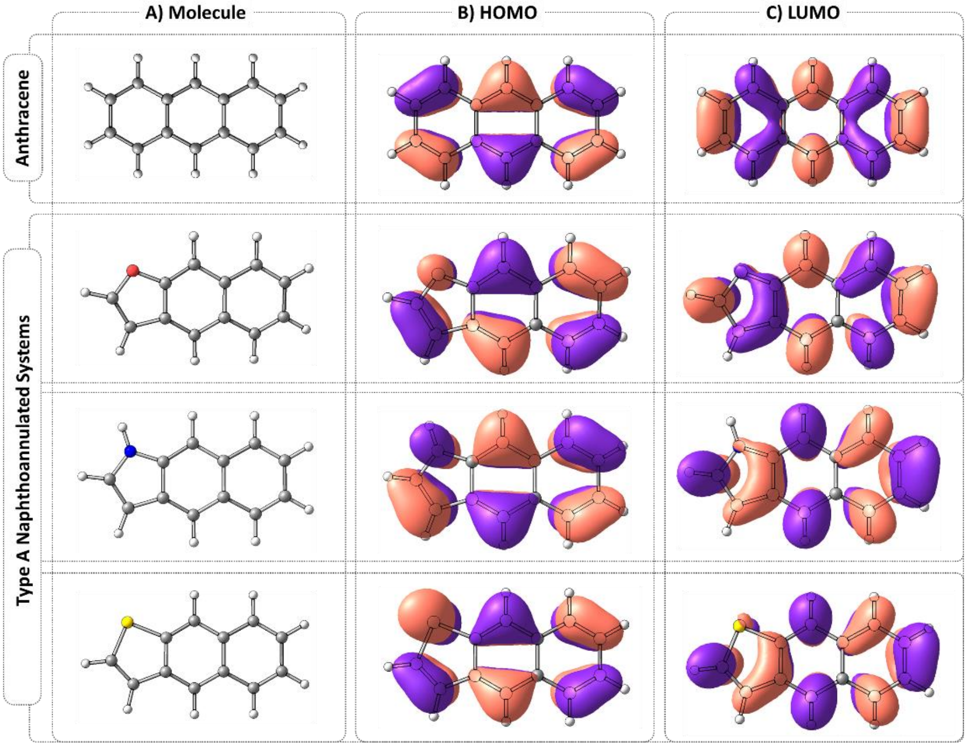

Figure S18: *Frontier molecular orbitals of anthracene and Type A tricyclic derivatives of furan, pyrrole, and thiophene.*

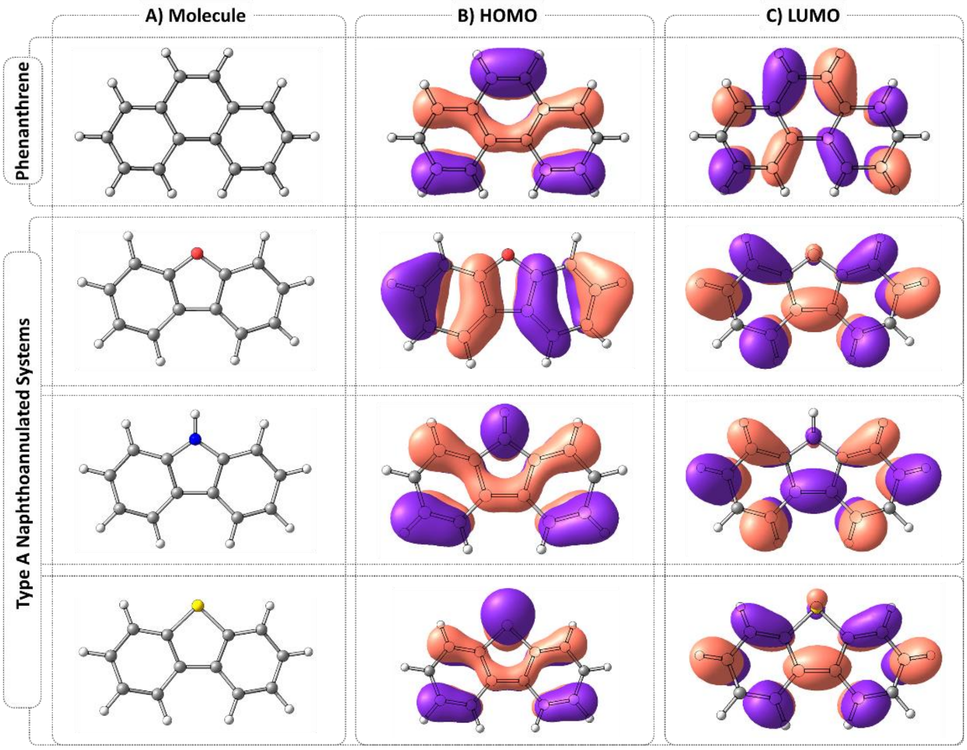

__Figure S19:__ Frontier molecular orbitals of phenanthrene and Type A tricyclic derivatives of furan, pyrrole, and thiophene.

For the Type B annulation, the situation is somewhat different. Nguyen *et al.*[7] proposed an analysis that is applicable to these cases. The authors showed that the HOMOs of five-membered heterocycles do not have a coefficient on the heteroatom, whereas the LUMOs feature a destabilizing interaction between the heteroatom lone pair and the π* of the 1,3-butadiene component of the ring. As shown in our calculations of the Type B benzannulated derivatives (**Figure S20−Figure S21**), this description is true for the bicyclic and tricyclic molecules, as well. According to this MO description, this causes the five-membered heterocycles to raise the LUMO while leaving the HOMO unchanged, which is in full agreement with our data for these systems. The authors also showed that the magnitude of the destabilizing interaction is linked to the aromaticity of the heterocycle. They quantified this effect with natural bonding orbital calculations and showed that pyrrole has by far the strongest destabilizing interaction,[7] which explains why it shifts the distribution much more than furan and thiophene. The fact that the pyrrole region also loses the linear shape that the cc-PBHs (and thiophene- and furan-derivatives) have suggests that the properties of the pyrrole-containing cc-hPASs are much more sensitive to variations in structure than their furan- and thiophene-containing counterparts.

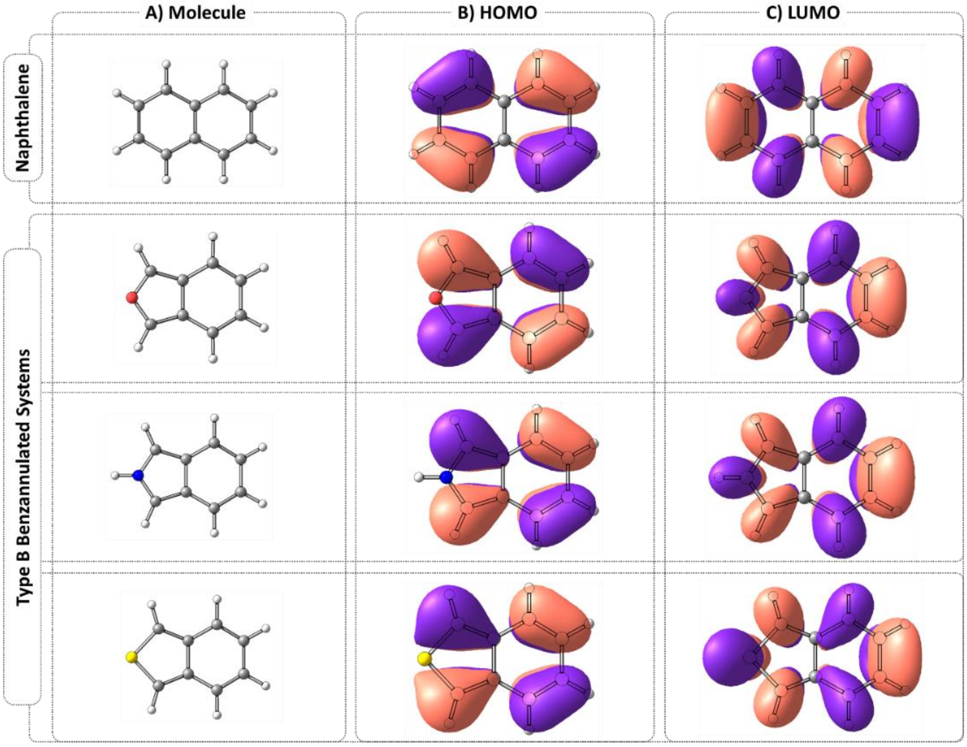

Figure S20: *Frontier molecular orbitals of naphthalene and Type B bicyclic derivatives of furan, pyrrole, and thiophene.*

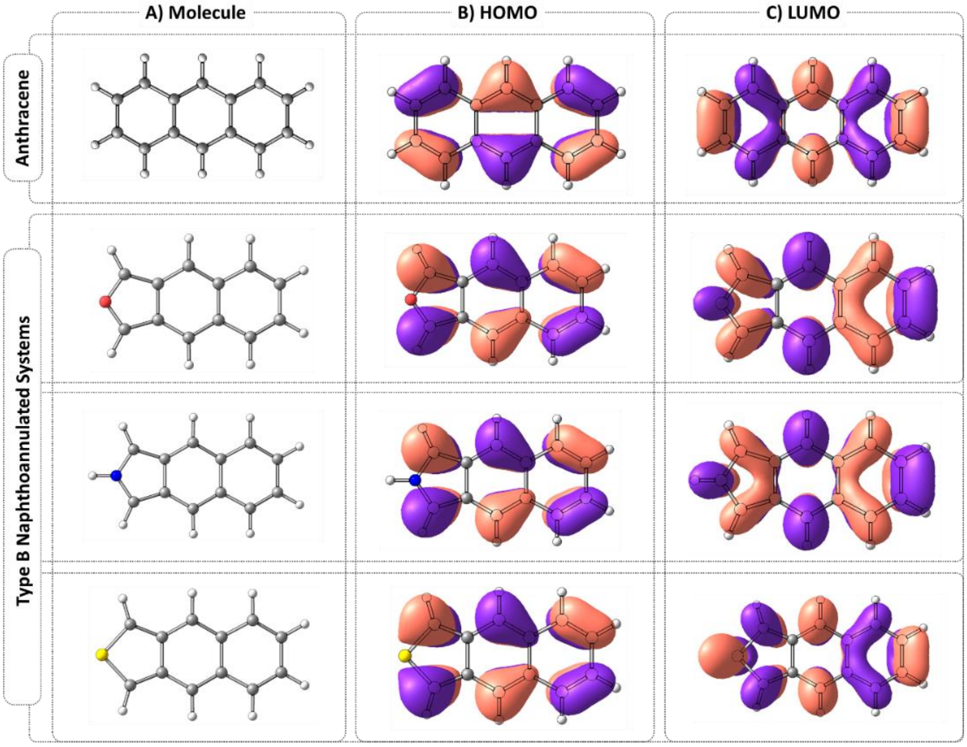

Figure S21: *Frontier molecular orbitals of anthracene and Type B tricyclic derivatives of furan, pyrrole, and thiophene.*

6.3. Antiaromatic boron-containing rings

The antiaromatic B-containing building blocks in our library are 1,4-dihydrodiborinine and borole.

We begin with the 6MR, 1,4-dihydrodiborinine. Once again, a comparison to the parent cc-PBHs is instructive (**Figure S22**). This comparison shows that the HOMOs of the B-containing systems look like the HOMO − 1 parent systems (naphthalene and anthracene), and the LUMOs of the B-containing systems look like the HOMOs of the respective of the parent systems (albeit with markedly larger coefficients on the B atom). These similarities suggest that the electronic systems remain quite similar, but simply contain 2 π-electrons fewer than the parent cc-PBHs. This also explains the extreme lowering of the HOMO and LUMO levels we observed for 1,4-dihydrodiborinine containing cc-hPASs, despite the lack of a coefficient on the B in the HOMO.

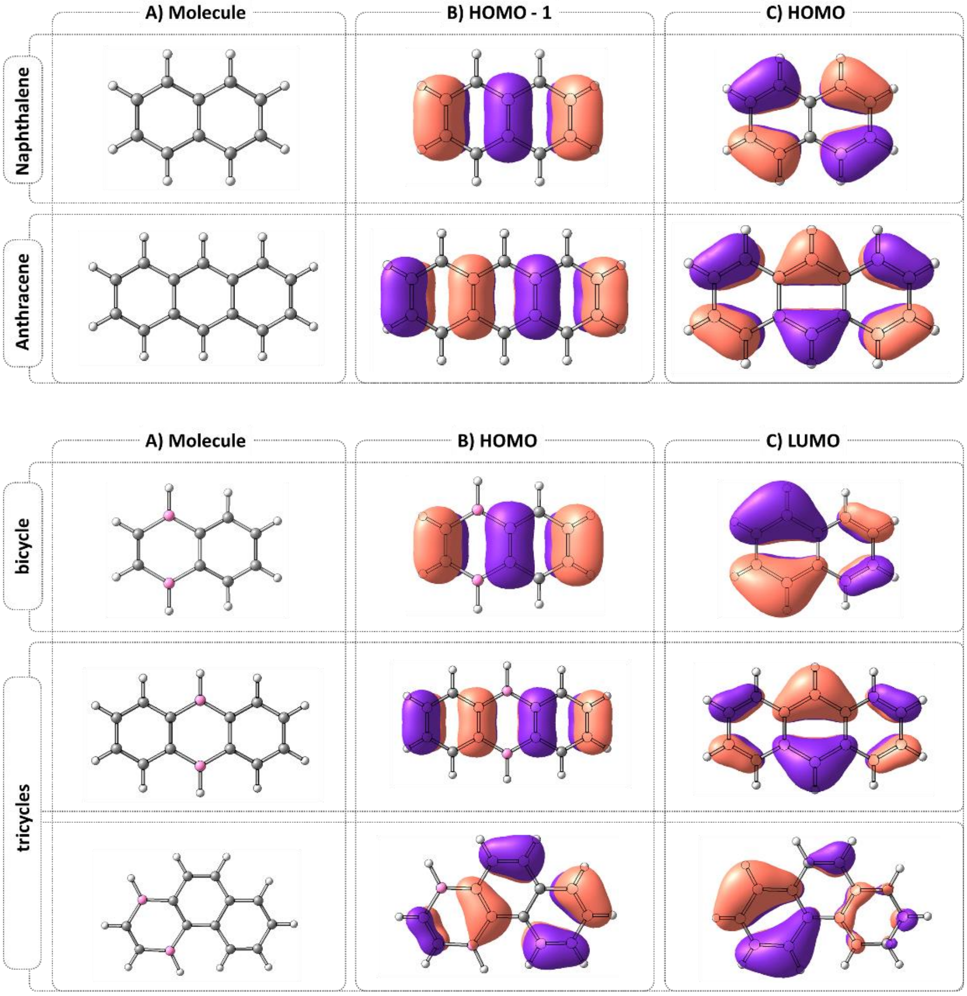

__Figure S22:__ Frontier molecular orbitals of naphthalene and anthracene (top), and bicyclic and tricyclic derivatives of 1,4-dihydrodiborinine-containing systems (bottom).

For the borole-containing systems, we once again differentiate between Type A and Type B cases (**Figure S23**). The Type A borole-containing molecules look extremely similar to the 1,4-dihydrodoborinine cases discussed above. Just as with those derivatives, the HOMO and LUMO of the borole-containing systems look like the HOMO − 1 and HOMO of naphthalene and anthracene, respectively. This, again, explains the lowering of both the HOMO and LUMO that our data-driven analysis revealed for these types of cc-hPASs.

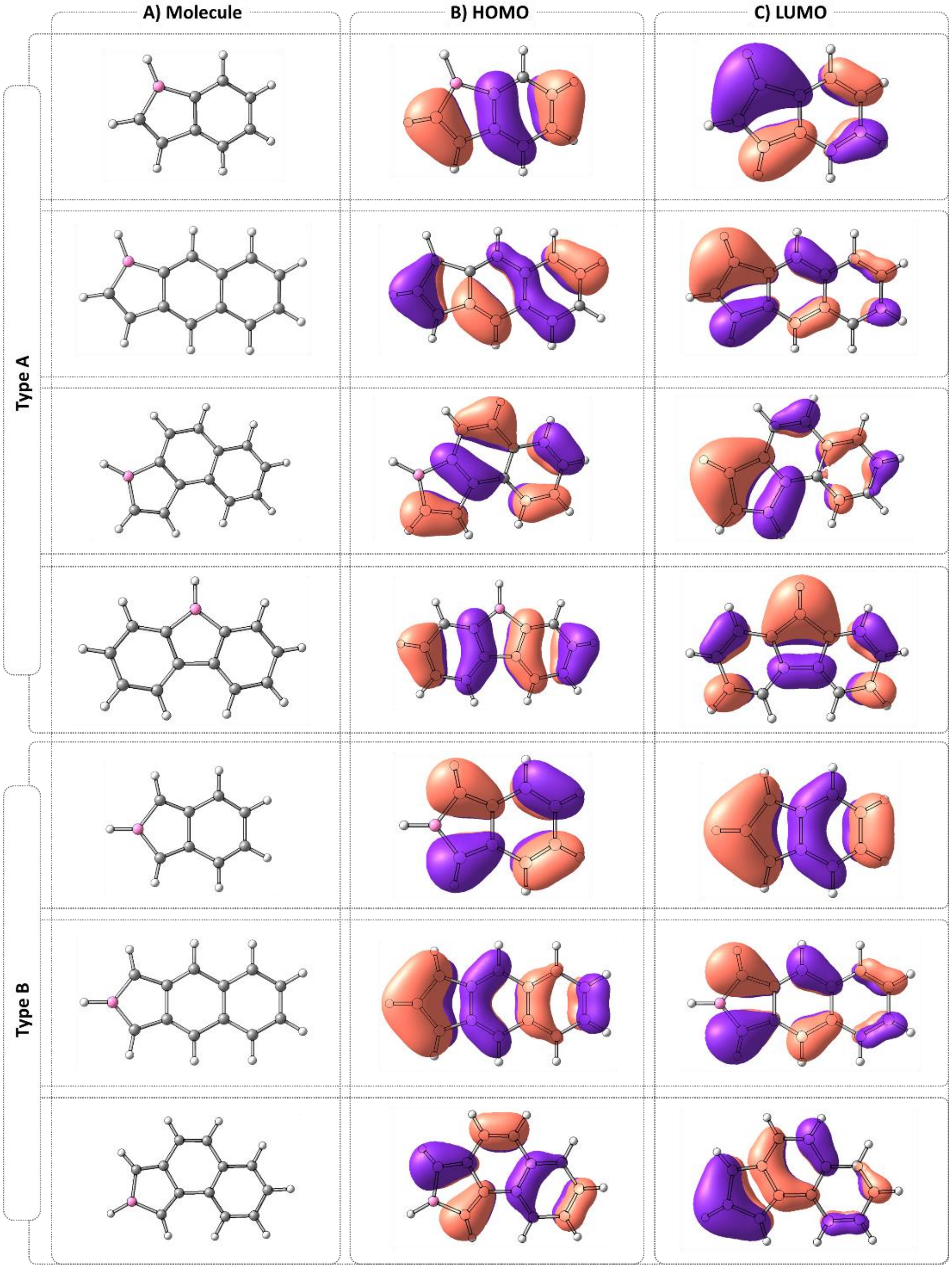

***Figure S23:** Frontier molecular orbitals of bicyclic and tricyclic derivatives of borole-containing systems, separated into Type A and Type B cases.*

The Type B cases are somewhat less straightforward to interpret. The bicyclic system (**Figure S23**) shows similarities to the Type B cases of the aromatic 5MRs. Here, as well, there is no coefficient on the B atom in the HOMO, and a large coefficient on the B atom in the LUMO. As opposed to the aromatic 5MRs, in which the lone pair has a destabilizing interaction, the empty *p* orbital of the B has a stabilizing interaction in the LUMO, which explains its LUMO-lowering effect. The orbitals of the angular tricyclic system are similar to the bicyclic case; the additional ring does not change the frontier MOs for the benzoborole component.

The linear tricyclic system shows a different behavior: the HOMO and LUMO appear to flip, as compared to the bicyclic analogue. This could indicate that the stabilizing effect in the LUMO becomes strong enough to lower this orbital even more than the HOMO. For this molecule, we also note that there are similarities between the HOMO and the HOMOs of the Type A case and between the LUMO and the LUMOs of the Type A cases. In other words, the HOMO is reminiscent of the HOMO − 1 of anthracene and the LUMO is reminiscent of the HOMO of anthracene.

7. References

(1) Winkler, M.; Houk, K. N. Nitrogen-Rich Oligoacenes: Candidates for n-Channel Organic Semiconductors. *J. Am. Chem. Soc.* **2007**, *129* (6), 1805–1815. https://doi.org/10.1021/ja067087u.

(2) Zhang, S.-F.; Chen, X.-K.; Fan, J.-X.; Guo, J.-F.; Ren, A.-M.; Li, Y.-W. Understanding the Effects of the Number of Pyrazines and Their Positions on Charge-Transport Properties in Silylethynylated N-Heteropentacenes. *J. Mol. Model.* **2014**, *20* (11), 2502. https://doi.org/10.1007/s00894-014-2502-3.

(3) Chen, X.-K.; Guo, J.-F.; Zou, L.-Y.; Ren, A.-M.; Fan, J.-X. A Promising Approach to Obtain Excellent N-Type Organic Field-Effect Transistors: Introducing Pyrazine Ring. *J. Phys. Chem. C* **2011**, *115* (43), 21416–21428. https://doi.org/10.1021/jp206617e.

(4) Bunz, U. H. F.; Engelhart, J. U.; Lindner, B. D.; Schaffroth, M. Large N-Heteroacenes: New Tricks for Very Old Dogs? *Angew. Chem. Int. Ed.* **2013**, *52* (14), 3810–3821. https://doi.org/10.1002/anie.201209479.

(5) Chen, Y.; Shen, L.; Li, X. Effects of Heteroatoms of Tetracene and Pentacene Derivatives on Their Stability and Singlet Fission. *J. Phys. Chem. A* **2014**, *118* (30), 5700–5708. https://doi.org/10.1021/jp503114b.

(6) Momicchioli, F.; Rastelli, A. On the Benzo-Derivatives of Five-Membered Heterocycles. Theoretical Treatment and UV Spectra. *J. Mol. Spectrosc.* **1967**, *22* (1), 310–324. https://doi.org/10.1016/0022-2852(67)90178-6.

(7) Delaere, D.; Nguyen, M. T.; Vanquickenborne, L. G. Influence of Building Block Aromaticity in the Determination of Electronic Properties of Five-Membered Heterocyclic Oligomers. *Phys. Chem. Chem. Phys.* **2002**, *4* (9), 1522–1530. https://doi.org/10.1039/B109008A.
